# Creation and preclinical evaluation of genetically attenuated malaria parasites arresting growth late in the liver

**DOI:** 10.1038/s41541-022-00558-x

**Published:** 2022-11-04

**Authors:** Blandine Franke-Fayard, Catherin Marin-Mogollon, Fiona J. A. Geurten, Séverine Chevalley-Maurel, Jai Ramesar, Hans Kroeze, Els Baalbergen, Els Wessels, Ludivine Baron, Valérie Soulard, Thomas Martinson, Maya Aleshnick, Antonius T. G. Huijs, Amit K. Subudhi, Yukiko Miyazaki, Ahmad Syibli Othman, Surendra Kumar Kolli, Olivia A. C. Lamers, Magali Roques, Rebecca R. Stanway, Sean C. Murphy, Lander Foquet, Diana Moita, António M. Mendes, Miguel Prudêncio, Koen J. Dechering, Volker T. Heussler, Arnab Pain, Brandon K. Wilder, Meta Roestenberg, Chris J. Janse

**Affiliations:** 1 Malaria Research Group, Department of Parasitology, Leiden University medical Center, Leiden, The Netherlands; 2 Department of Medical Microbiology, Leiden University Medical Center, 2300 RC Leiden, the Netherlands; 3 Sorbonne Université, Institut National de la Santé et de la Recherche Médicale (INSERM), Centre National pour la Recherche Scientifique (CNRS), Centre d’Immunologie et des Maladies Infectieuses, CIMI, Paris, France; 4 Vaccine and Gene Therapy Institute, Oregon Health and Science University, Beaverton, OR 97006 USA; 5 TropIQ Health Sciences, Nijmegen, the Netherlands; 6 Pathogen Genomics Laboratory, Biological and Environmental Sciences and Engineering (BESE) Division, King Abdullah University of Science and Technology (KAUST), Thuwal, Kingdom of Saudi Arabia; 7 Universiti Sultan Zainal Abidin, Faculty of Health Sciences, Terengganu, Malaysia; 8 Institute of Cell Biology, University of Bern, Bern, Switzerland; 9 Department of Laboratory Medicine and Pathology, University of Washington, Seattle, WA USA; 10 Yecuris Corporation, Tualatin, OR USA; 11 Instituto de Medicina Molecular João Lobo Antunes, Faculdade de Medicina, Universidade de Lisboa, Lisboa, Portugal; 12 International Institute for Zoonosis Control, Hokkaido University, N20 W10 Kita-ku, Sapporo, Japan; 13 Department of Infectious Diseases, Leiden University Medical Center, Leiden, The Netherlands; 14 Present Address: Department of Cellular Architecture Studies, Institute of Tropical Medicine, Nagasaki University, Nagasaki, Japan; 15 Present Address: Center for Global Health and Infectious Diseases Research, College of Public Health, University of South Florida, Tampa, FL USA; 16 Present Address: Multidisciplinary Center for Infectious Diseases (MCID), University of Bern, Bern, Switzerland

**Keywords:** Diseases, Malaria

## Abstract

Whole-sporozoite (WSp) malaria vaccines induce protective immune responses in animal malaria models and in humans. A recent clinical trial with a WSp vaccine comprising genetically attenuated parasites (GAP) which arrest growth early in the liver (PfSPZ-GA1), showed that GAPs can be safely administered to humans and immunogenicity is comparable to radiation-attenuated PfSPZ Vaccine. GAPs that arrest late in the liver stage (LA-GAP) have potential for increased potency as shown in rodent malaria models. Here we describe the generation of four putative *P. falciparum* LA-GAPs, generated by CRISPR/Cas9-mediated gene deletion. One out of four gene-deletion mutants produced sporozoites in sufficient numbers for further preclinical evaluation. This mutant, *Pf*Δ*mei2*, lacking the *mei2-like RNA* gene, showed late liver growth arrest in human liver-chimeric mice with human erythrocytes, absence of unwanted genetic alterations and sensitivity to antimalarial drugs. These features of *Pf*Δ*mei2* make it a promising vaccine candidate, supporting further clinical evaluation. *Pf*Δ*mei2* (GA2) has passed regulatory approval for safety and efficacy testing in humans based on the findings reported in this study.

## Introduction

Whole-sporozoite (WSp) malaria vaccines can induce strong protective immune responses in animal models of malaria and in humans^1–4^. WSp vaccines consist of whole parasites, i.e., metabolically active *P. falciparum* sporozoites (PfSPZ) that have the ability to migrate to and infect the human liver, but cannot transform into the symptomatic blood stage, and as such do not cause disease. The most advanced WSp vaccine candidate, the PfSPZ Vaccine, employs sporozoites that have been attenuated by radiation^5^. These sporozoites enter hepatocytes but are unable to replicate and thus abort development early in the liver. The PfSPZ Vaccine, consisting of cryopreserved, vialed radiation-attenuated sporozoites and produced by the biotech company Sanaria, Inc. (US), has entered phase 3 clinical development^6,7^. Sporozoite attenuation by genetic modification rather than by radiation, offers the advantage of a more homogeneous product, increased biosafety for sporozoite production, and potentially increased potency^8,9^. In a recent clinical study, human volunteers were immunized by intravenous injection with multiple doses of cryopreserved, vialed sporozoites of either the PfSPZ Vaccine or a WSp vaccine, consisting of genetically-attenuated parasites (GAP) that arrest growth soon after invasion of hepatocytes (early-arresting GAP; EA-GAP)^10,11^. This study showed that the EA-GAP vaccine, termed PfSPZ-GA1, was safe and induced immune responses that were comparable to those induced by the radiation-attenuated PfSPZ Vaccine^10^. Multiple clinical studies with the PfSPZ Vaccine in healthy volunteers from non-endemic areas (e.g., without prior exposure to malaria), have shown that this vaccine can induce levels of protection that are higher than those achieved with various subunit malaria vaccines (protein, peptide, or DNA-based)^3,12,13^. However, high sporozoite numbers are needed to achieve sufficient levels of efficacy. Moreover, the efficacy of the PfSPZ Vaccine appears to be lower in people living in areas of malaria endemicity compared to people with no previous history of malaria infection^14,15^, creating a need for highly potent WSp vaccines to achieve the 75% protection against clinical disease for >1 year as targeted in the malaria vaccine technology roadmap (https://www.malariavaccine.org/malaria-and-vaccines/malaria-vaccine-roadmap). Chemo-attenuated PfSPZ immunization studies suggest that the WSp vaccine efficacy may be improved by delaying the growth arrest in the liver and thereby broadening the antigen repertoire^16^. A study using a mouse malaria GAP has shown that attenuated sporozoites that arrest growth late in the liver (late-arresting GAP; LA-GAP) through deletion of a gene encoding a protein involved in fatty acid synthesis (Fabb/f), induced stronger protective immune responses than immunization with EA-GAP sporozoites^17^. The increased potency can most likely be explained by the exposure to an increased (liver- and blood-stage) antigen repertoire and/or biomass of LA-GAP liver-stages compared to those of EA-GAP. Unfortunately, removal of several fatty acid synthesis genes from the *P. falciparum* genome, results in the arrest of parasite growth in the mosquito preventing sporozoite formation^18,19^. These genes are therefore unsuitable targets for creating a *P. falciparum* LA-GAP for human use. To generate a *P. falciparum* LA-GAP, we selected in this study four additional genes (*palm, hcs1, cbr, mei2*) with a putative specific role in *P. falciparum* late liver-stage development, based on published phenotypes of rodent malaria mutants that lack the orthologous genes. We report the creation and analysis of four *P. falciparum* gene-deletion mutants that lack the *palm, hcs1, cbr,* or *mei2* gene. We show that three gene-deletion mutants did not show the expected phenotype of sporozoite production, essential for further preclinical evaluation. Only the *P. falciparum* mutant lacking the *mei2* gene (*Pf*Δ*mei2*) produced infective sporozoites that arrest growth late during development in the liver. We report results of preclinical evaluation of *Pf*Δ*mei2*, involving evaluation of late liver growth-arrest in cultured primary human hepatocytes and in chimeric mice with humanized liver and human red blood cells, genotype analysis by whole-genome sequencing and in-vitro drug-sensitivity testing of blood stages. Based on these preclinical studies, *Pf*Δ*mei2* has passed regulatory approval for clinical evaluation of safety and efficacy as an LA-GAP vaccine (GA2).

## Results

### Selection of genes with a role during late liver-stage *Plasmodium* development

Gene-deletion studies have identified multiple proteins that play a role during late liver-stage development of rodent malaria parasites. Table 1 shows liver-stage growth phenotypes of published gene-deletion mutants that display a normal, wild-type-like blood-stage and sporozoite development but present defects in late liver-stage development. Most mutants do not display full growth arrest in the liver as revealed by ‘break-through’ blood infections, although with a (strong) increase in prepatency. Deletion of genes encoding apicoplast-located proteins involved in fatty acid synthesis results in the strongest attenuation phenotype, specifically in *P. yoelii*, with complete growth arrest. However, FASII-pathway genes, involved in the transformation of Acetyl-CoA into Acyl-ACP, are unsuitable targets for generation of *P. falciparum* LA-GAP because this pathway is essential for formation of viable sporozoites^18^. It can be assumed that genes encoding the apicoplast-located proteins of the pyruvate dehydrogenase (PDH) complex, which provides Acetyl-CoA, are also essential for *P. falciparum* sporozoite formation. Therefore, we excluded FASII-pathway genes for generation of *P. falciparum* LA-GAP. Four genes with a previously reported strong attenuation phenotype, *palm, hcs1, cbr*, and *mei2*, were selected for creating *P. falciparum* LA-GAPs. This selection was based on the absence of blood infections and/or compelling extension of the prepatent period after infection of mice with gene-deletion mutant sporozoites, while these sporozoites developed into late liver-stages in cultured hepatocytes (Table 1). The *P. berghei* PALM protein, whose exact function is unknown but plays a role during the final steps of *P. berghei* liver-stage merozoite formation^20^, has been shown to localize to the apicoplast^20^. HCS1, an ATP-dependent ligase that catalyzes biotin binding to biotin carboxylase, plays a role in adequate formation of *P. berghei* liver-stage merozoites^21^ and is expressed in the cytoplasm of liver-stages^21^. For CBR, a putative cytochrome-b5 oxidoreductase, a location in the food vacuole of blood-stages has been reported^22^. *P. berghei* parasites lacking CBR, showed a slight reduction in the size of mature liver-stages and in detachment of cultured hepatocytes containing maturing liver-stages^23^. The MEI2 protein is a member of a family of RNA-binding proteins containing an RNA recognition motif^24^. *P. yoelii* parasites lacking MEI2 develop into late liver schizonts but have a complete attenuation phenotype in highly susceptible BALB/cByJ mice infected with 50,000 sporozoites^24^ and only occasional breakthrough blood-infections were observed when >200,000 sporozoites were inoculated^25^. We confirmed this late liver-stage growth arrest and the strong attenuation phenotype in *P. berghei* by creating and characterizing *P. berghei* gene-deletion mutants lacking the *mei2* gene (*Pb*Δ*mei2;* Supplementary Fig. 1). We also observed occasional breakthrough blood-infections when mice were inoculated with high numbers (2 × 10^5^) of *Pb*Δ*mei2* sporozoites. In one experiment we infected mice with 2 × 10^5^ sporozoites of a *Pb*Δ*mei2* line (*Pb*Δ*mei2-breakthrough-a)* that was collected from mice with a breakthrough blood-infection after the first infection with *Pb*Δ*mei2* sporozoites. In this experiment we found that the percentage of mice with a ‘second’ breakthrough blood infection was comparable to that of mice with a ‘first’ breakthrough infection and the mice that became positive had again a strongly prolonged prepatent period, i.e., 5 of the 11 mice did develop a blood stage infection with a prepatent period of 9-10 days, whereas mice infected with wild-type parasites became blood-stage positive at day 4 or 5. These observations strongly indicate that the *Pb*Δ*mei2-breakthrough-a* parasites are not derived from parasites that had permanently switched to an efficient and Mei2-independent mechanism of liver stage development (Supplementary Fig. 1).

**Table 1:** Published rodent malaria gene-deletion mutants with a ‘late-liver stage’ growth phenotype.

berghei/yoelii gene ID	falciparum gene ID	product	blood infection	Extended prepatency	Ref.
PBANKA_1125100	PF3D7_0626300	3-oxoacyl-acyl-carrier protein synthase I/II (FabB/FabF)	yes	yes	^70^
PY17X_1126500	PF3D7_0626300	3-oxoacyl-acyl-carrier protein synthase I/II (FabB/FabF)	no	-	^71^
PBANKA_1229800	PF3D7_0615100	enoyl-acyl carrier reductase (ENR,FabI)	yes	yes	^72^
PY17X_1342900	PF3D7_1323000	beta-hydroxyacyl-ACP dehydratase (FabZ)	no	-	^71^
PBANKA_1410500	PF3D7_1312000	malonyl CoA-acyl carrier protein transacylase (FabD)	no	-	^23^
PBANKA_0308200	PF3D7_0211400	beta-ketoacyl-ACP synthase III (FabH)	yes	yes	^23^
PBANKA_0823800	PF3D7_0922900	3-oxoacyl-[acyl-carrier-protein] reductase (FabG)	no	-	^23^
PY17X_0715100	PF3D7_0815900	dihydrolipoyl dehydrogenase, apicoplast (PDH E3)	no	-	
PBANKA_0923800	PF3D7_1124500	pyruvate dehydrogenase E1 component subunit	no	-	^73^
		alpha (PDH E1α)			
PY17X_0934900	PF3D7_1114800	glycerol-3-phosphate dehydrogenase, putative (apiG3PDH)	no	-	^74^
PBANKA_0923800	PF3D7_1114800	glycerol-3-phosphate dehydrogenase, putative (apiG3PDH)	yes	yes	^73^
PY17X_1418400	PF3D7_1318200	glycerol-3-phosphate 1-O-acyltransferase (apiG3PAT)	no	-	^74^
PBANKA_0820900	PF3D7_0920000	long chain fatty acid elongation enzyme, putative (ELO3, ELO-A)	no	-	^23^
PBANKA_1357500	PF3D7_1344600	lipoyl synthase (LipA)	yes	yes	^23^
PBANKA_0707000	PF3D7_0823600	lipoate-protein ligase B (LipB)	yes	yes	^21^
PBANKA_1143400	PF3D7_1367500	NADH-cytochrome b5 reductase, putative (CBR)	yes	yes	^23^
PBANKA_0101100	PF3D7_0602300	liver merozoite formation protein (PALM)	Yes	Yes	^20^
PBANKA_0511000	PF3D7_1026900	biotin--protein ligase 1 (HCS1)	yes	yes	^21,23^
PY17X_1123700	PF3D7_0623400	MEI2-like RNA-binding protein (MEI2)	Yes	Yes	^24^
PBANKA_0214400	PF3D7_0730300	a transcription factor with AP2 domain(s) (AP2-L)	Yes	Yes	^75^
PBANKA_1024600	PF3D7_1418100	putative liver stage protein 1 (LISP1)	Yes	Yes	^76^
PBANKA_1003000	PF3D7_0405300	liver-specific protein 2 (LISP2; sequestrin)	Yes	Yes	^77,78^
PBANKA_0506500	PF3D7_1022300	ZIP domain-containing protein (ZIPCO)	Yes	Yes	^79^
PBANKA_0505000	PF3D7_1020800	dihydrolipoamide acyltransferase component E2	yes	yes	^23^
PBANKA_1436200	PF3D7_1221000	histone-lysine N-methyltransferase, H3 lysine-4 specific	yes	yes	^23^
PBANKA_0304800	PF3D7_0207400	serine repeat antigen 7	yes	yes	^80^
PBANKA_0404100	PF3D7_0305600	DNA-(apurinic or apyrimidinic site) endonuclease	yes	yes	^81^
PBANKA_1021500	PF3D7_1421900	copper transporter, putative (CTR2)	yes	yes	^82^

Genes 1–7: Apicoplast-located proteins, involved in fatty acid synthesis (FAS II)–transformation of Acetyl-CoA into Acyl-ACP; Genes 8–12: Apicoplast-located proteins of the pyruvate dehydrogenase (PDH) complex, providing acetyl-CoA for fatty acid synthesis; Genes 13–17: (Putative) apicoplast-located proteins: not involved in the transformation of Acetyl-CoA into Acyl-ACP; Genes 16–19: genes selected for gene-deletion in *P. falciparum.*

### *P. falciparum* mutants lacking the genes *palm*, *hcs1* or *cbr* do not produce viable sporozoites

**Fig. 1:**
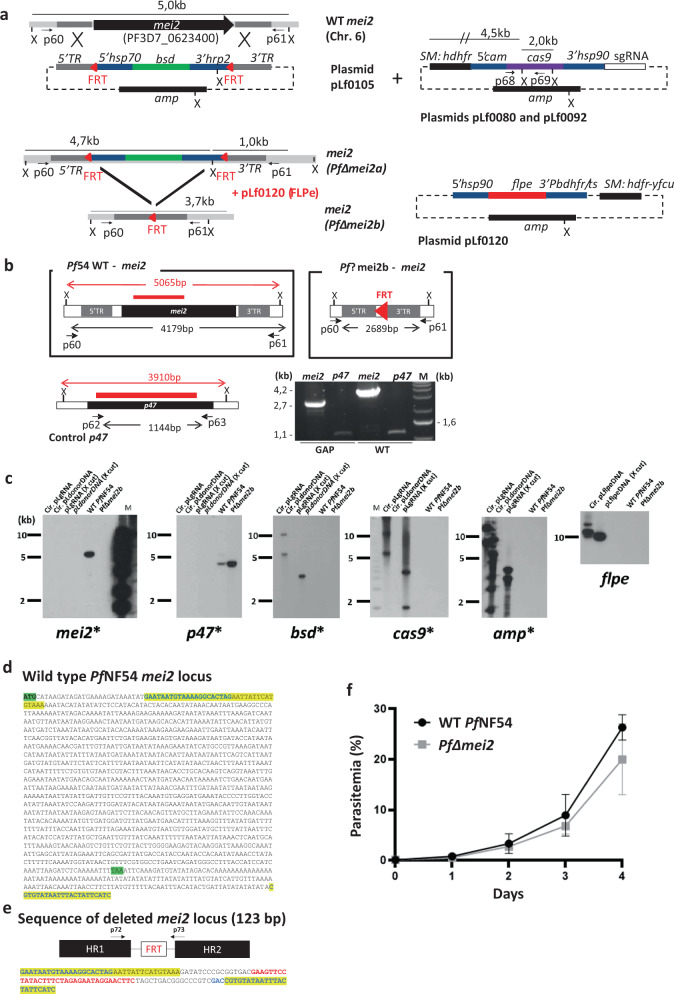
Generation, genotyping, and blood-stage growth of *Pf*Δ*mei2* (LA-GAP, GA2). **a** Left: the *mei2* (PF3D7_ 0623400) genomic locus on chromosome 6 (Chr. 6) of wild-type *Pf* NF54 (WT *Pf* NF54) and *Pf*Δ*mei2* parasites before (*Pf*Δ*mei2a*) and after (*Pf*Δ*mei2b*) FLPe-mediated removal of the *blasticidin-S-deaminase* (*bsd*) selectable marker (SM). The donor plasmid pLf0105 to delete *mei2* contains the *bsd* SM, flanked by two *frt* sites (red triangles) and *mei2* targeting sequences (5′ TR and 3′ TR) for double cross-over integration. Primer pairs p60/p61 and PCR fragment size for diagnostic PCR are indicated (**b**); X (*Xmn*I): restriction site used for Southern blot analyses (**c**). *hsp70*, *heat shock protein 90; hrp2, histidine-rich protein II; amp, ampicillin*. Right top: sgRNA plasmids (pLf0080, pLf0092) containing the human *dihydrofolate reductase-thymidylate synthase* (h*dhfr*) SM and the *cas9* expression cassette. *Cam, calmodulin*. PCR primers (p68/p69) to amplify part of *cas9*, sizes of the sgRNA constructs after *Xmn*I (X) digestion and *mei2* and *cas9* probes are indicated (**c**). Right bottom: construct pLf0120 with the h*dhfr-*y*fcu* SM and the *flpe* expression cassette. pb*dhfr/ts,: P. berghei bifunctional dihydrofolate reductase-thymidylate synthase, putative*. See Supplementary Table 1 for primers details. **b** WT *Pf* NF54 and *Pf*Δ*mei2b* genomic loci and the control gene *p47* (coding sequence shown as black boxes). Shown are the 5′ and 3′ *mei2* targeting regions (5′TR and 3′TR), used construct pLf0105 (**a**) and the *frt* site. PCR primers (in black) for amplifying *mei2* (p60/p61) and *p47* (p62/63), expected sizes of the full length *mei2* and *p47* genes and size of *mei2* locus after *mei2* deletion and removal of *bsd* SM cassette are shown. X (*Xmn*I): restriction site used for Southern analysis (**c**). DNA probes used in Southern analyses (**c**) and sizes of digested DNA fragments recognized by the probes (*mei2* and *p47*) are shown (in red). Red triangle: the 34 bp *frt* site in the *Pf*Δ*mei2*b genome after removal of the *bsd* SM cassette. PCR (right lower panel) analysis of WT *Pf* NF54 and *Pf*Δ*mei2*b genomic DNA confirms *mei2* deletion (control: amplification of *p47*). Primer pairs: p60/61 for *mei2* and p62/p63 for *p47*. See Supplementary Table 1 for primer details). M, molecular weight marker; 1 kb DNA ladder (Invitrogen). **c** Southern analysis of restricted genomic DNA from WT *Pf* NF54, *Pf*Δ*mei2*b, and plasmids used to delete *mei2* (DNA digested with *Xmn*I (X)). DNA-samples/lanes: (i) circular sgRNA plasmids (Cir.pLgRNA); (ii) circular donor DNA plasmid pLf0105 (pLΔ*mei2*); (iii) *XmnI*-digested sgRNA plasmid (pLgRNA-X cut); (iv) *Xmn*I-digested donor DNA plasmid; v) genomic WT *Pf* NF54 DNA; (vi) genomic *Pf*Δ*mei2*b DNA. Probes: part of *mei2*, *p47* (control), *cas9*, *bsd*, *amp* and *flpe* (see **a** and **b** for probe location and expected fragment sizes). Hybridizations show correct *mei2* deletion and absence of *cas9, bsd, amp* and *flpe* in *Pf*Δ*mei2*b. M, molecular weight marker; 1 kb DNA ladder (Invitrogen) labeled on the sides of the gels. **d** Sequence of the WT *Pf*NF54 *mei2* locus. Yellow: sequences present in the disrupted *mei2* locus of *Pf*Δ*mei2* (**e**); Green: start and stop codon of *mei2*. **e** The *mei2* locus in the *Pf*Δ*mei2* genome after *mei2* deletion by integration of pLf0105 and FLPe-mediated removal of the *bsd* SM marker The *mei2* targeting regions (HR1 and HR2) for double cross-over integration and the *frt* site are shown. In addition, the sequence of the PCR fragment of the *mei2* locus is shown, amplified using primers p72/p73 (see Supplementary Table 1 for primer details). Yellow: sequences present in the *mei2* locus of WT *Pf*NF54 and *Pf*Δ*mei2*. Red: the 34 bp FRT sequence, flanked by 16 bp and 14 bp cloning restriction sites. **f** In vitro growth rate of *Pf*Δmei2 and WT *Pf* NF154 asexual blood stages. Parasitemia (%) during a 4-day culture period (mean and s.d. of three cultures). Error bars represent standard deviation.

To create *P. falciparum* LA-GAP, independent mutants lacking the *palm, hcs1, cbr,* or *mei2* genes (*Pf*Δ*palm, Pf*Δ*hcs1*, *Pf*Δ*cbr, Pf*Δ*mei2*) were generated using established methods of CRISPR/Cas9 gene-editing^26,27^. Correct deletion of the genes was confirmed by diagnostic PCR and/or Southern blot analysis of digested genomic DNA (Supplementary Figs. 2–4; Fig. 1). These four mutants showed blood-stage growth rates comparable to wild-type *P. falciparum* NF54 (WT *Pf*NF54) parasites. In vitro gametocyte production and oocyst numbers in *An. stephensi* mosquitoes fed with cultured gametocytes were in the range of those of WT *Pf*NF54 (Supplementary Figs. 2–4; Fig. 1; Table 2). However, in mosquitoes infected with the *Pf*Δ*palm, Pf*Δ*hcs1,* and *Pf*Δ*cbr* mutants, salivary gland sporozoites were either absent or their numbers were highly reduced. Midgut/hemocoel sporozoites were also absent or strongly reduced and most oocysts did not show clear signs of sporozoite formation, presenting an ‘empty’ or vacuolated appearance (Supplementary Fig. 5). These observations indicate a role of these three proteins in adequate sporozoite formation inside oocysts. Only *Pf*Δ*mei2* mutants produced numbers of salivary gland sporozoites comparable to those observed for WT *Pf*NF54 (Table 2).

**Table 2:** Gametocyte, oocyst, and sporozoite production of four *P. falciparum* gene-deletion mutants and wild-type WT *Pf*NF54.

Lines	Stage V Gam. (%)^1^	Exflag./10^5^ RBC^2^	Infection rate (%)^3^	Oocyst no.^4^	Spor. no (×10^3^)^6^
	Average (s.d)	Average (s.d.)	Average (s.d.)	Average (s.d.)	Average (s.d.)
WT *Pf*NF54	2.04 (0.88)	804 (387)	66 (39)	92 (86)	53 (51)
	0.8–3.7 (11 exp.)	201–1459 (27 exp.)	68–100 (34 exp.)	21–247 (34 exp.)	4–155 (34 exp.)
PfΔpalm	2.85 (1.34)	1544 (116)	75 (18)	38 (27)	0
	1.9–3.8 (2 exp.)	1462–1626 (2 exp.)	56–93 (4 exp.)	19–64 (4 exp.)	(4 exp.)
PfΔcbr	1.6 (0.6)	440 (219)	53 (45)	65 (52)	0
	0.8–2.1 (4 exp.)	147–660 (9 exp.)	54–100 (13 exp.)	22–156 (13 exp.)	(11 exp.)
PfΔhcs1	2.6 (0.8)	459 (133)	92(14)	89 (42)	0
	1.8–3.3 (3 exp.)	365–554 (2 exp.)	76–100 (3 exp.)	63–138 (3 exp.)	(3 exp.)
PfΔmei2	2.1 (0.6)	288 (182)	73 (32)	63 (68)	31 (26)
	1.8–2.8 (3 exp.)	94–600 (9 exp.)	23–100 (14 exp.)	14–167 (13 exp.)	10–84 (9 exp.)

^1^Mean percentage of blood-stage parasites developing into gametocytes in vivo.

^2^Mean percentage of exflagellating males in vitro, 12–15 minutes after activation.

^3^percentage of infected mosquitoes at days 7–12 after feeding.

^4^Mean number of oocysts per mosquito (days 7–12).

^5^Mean number of sporozoites per mosquito (days 21).

s.d.: standard deviation. Second raw: range and number of experiments.

### *PfΔmei2* parasites develop into replicating liver stages but fail to produce red blood cell-infectious liver-stage merozoites

**Fig. 2:**
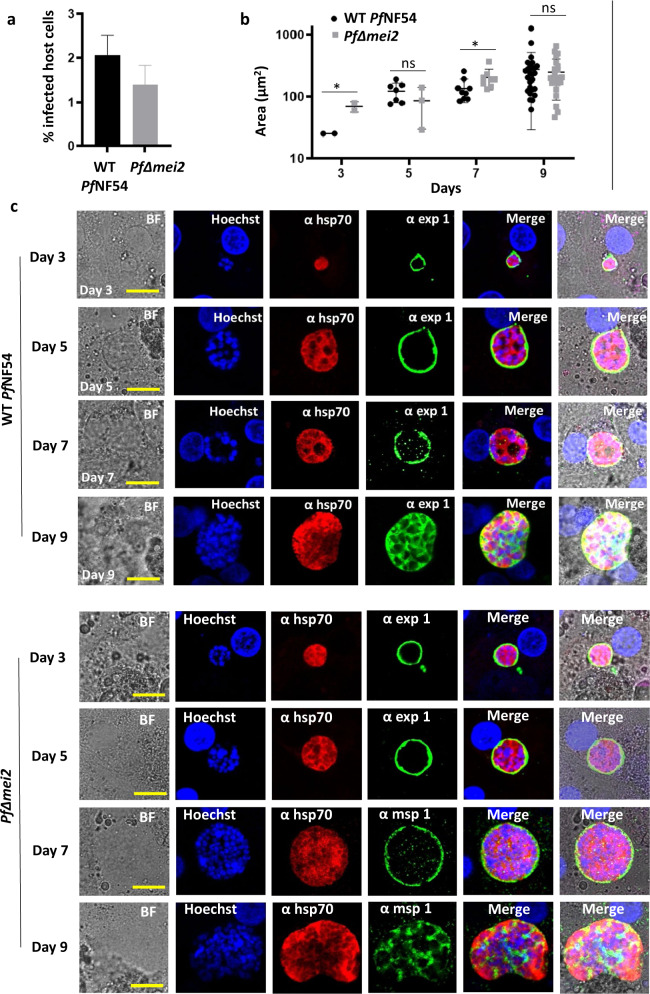
*Pf*Δ*mei2* liver-stage development in cultured human primary hepatocytes (BioIVT). **a** Percentage of hepatocytes infected with WT *Pf*NF54 and *Pf*Δmei2 at day 3 post infection (p.i.) (*p* = 0.002; unpaired Mann–Whitney test). **b** Liver-stage size on day 3, 5, 7, and 9 p.i. (3-20 parasites measured in two wells). The average of the parasite’s cytoplasm at its greatest circumference using HSP70-positive area (μm^2^), s.d. and significances values are shown (unpaired Mann–Whitney test: *p < 0.05; ns: not significant). **c** Representative confocal microscopy images of liver stages on days 3, 5, 7, and 9 p.i. Upper panel WT *Pf*NF54; lower panel *Pf*Δmei2. Fixed hepatocytes were stained with the following antibodies: rabbit anti-*Pf*HSP70 (α hsp70), mouse anti-*Pf*EXP1 (α exp1), and anti-*Pf*MSP1 (α msp1). Nuclei stained with Hoechst-33342. All pictures were recorded with standardized exposure/gain times; Alexa Fluor® 488 (green) 0.7 s; anti-IgG Alexa Fluor® 594 (red) 0.6 s; Hoechst (blue) 0.136 s; bright field (BF) 0.62 s (1× gain). Scale bar, 10 μm. Error bars represent standard deviation.

The growth arrest of *Pf*Δ*mei2* liver stages was investigated using primary human hepatocytes. First, *Pf*Δ*mei2* sporozoite infectivity and development was analyzed in vitro in cultures of cryopreserved primary human hepatocytes (from the company BioIVT) infected with isolated *Pf*Δ*mei2* and WT *Pf*NF54 sporozoites. A mean of 2,1% (s.d. 0.42) and 1.4% (s.d. 0.42) infected hepatocytes per well was observed for WT *Pf*NF54 WT and *Pf*Δ*mei2*, respectively, at day 3 post infection (p.i.) (Fig. 2a). During in vitro liver-stage development (day 3, 5, 7, and 9 p.i.) the size and light-microscopy morphology of *Pf*Δ*mei2* parasites was comparable to those of WT *Pf*NF54 parasites and a strong increase in parasite size (Fig. 2b) and nuclear content (Fig. 2c) was observed in both WT *Pf*NF54 and *Pf*Δ*mei2* parasites. Liver-stages of WT *Pf*NF54 and *Pf*Δ*mei2* liver stages displayed similar staining patterns of the cytoplasmic marker HSP70 and of the parasitophorous vacuole marker EXP1. At days 7 and 9 p.i., liver-stages of WT *Pf*NF54 and *Pf*Δ*mei2* exhibited comparable staining of the merozoite surface protein 1 (MSP1) (Fig. 2c). We then confirmed wild-type-like hepatocyte invasion and development into late liver-stages of *PfΔmei2* in cryopreserved primary human hepatocytes from the company Lonza (Supplementary Fig. 6) These analyses also showed that sporozoite infectivity and the size of *PfΔmei2* liver stages (at day 3, 5, 7, and 9) were comparable to those of WT *Pf*NF54 parasites. In addition, *PfΔmei2* and WT *Pf*NF54 liver stages displayed similar staining patterns with antibodies against the cytoplasmic marker HSP70 and the parasitophorous vacuole marker EXP1. All together, these results show that *Pf*Δ*mei2* sporozoites can effectively infect cultures of human hepatocytes and are able to develop into large, replicating liver-stages that express merozoite-specific antigens.

**Fig. 3:**
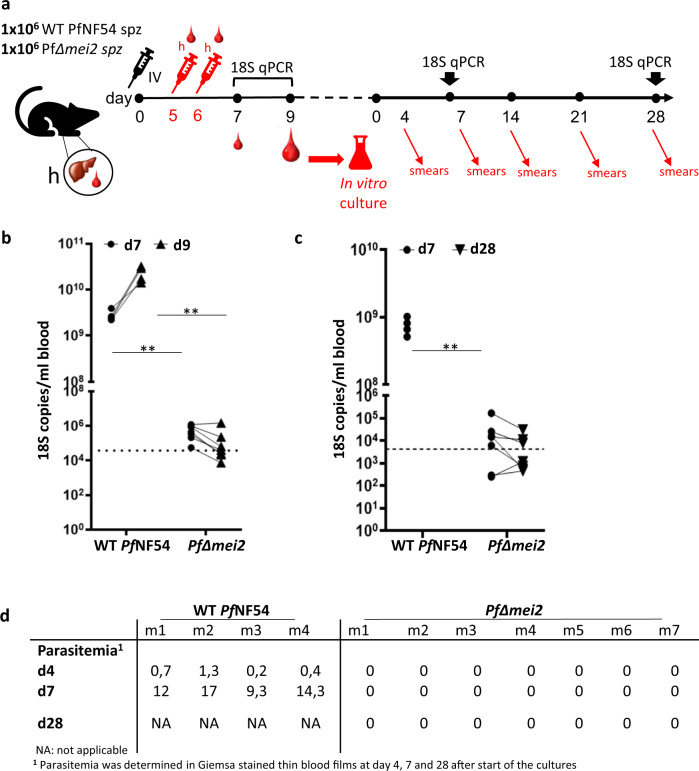
Development of *Pf*Δ*mei2* in FRG huHep mice containing human red blood cells. **a** Timeline of experiments in liver-chimeric mice where mice were injected intravenously (IV) with 1 × 10(6) sporozoites on day 0 and then with human red blood cells (h) on days 5 and 6 prior to emergence of blood-stage parasites. Blood samples were taken on days 7 and 9 for qRT-PCR with day 9 samples used for 28-day in vitro culture of blood stages with subsequent parasitaemia readout by microscopy and qRT-PCR. **b** 18 S qPCR analysis of blood samples from FRG huHep mice on day 7 and 9 after infection with 1 × 10(6) sporozoites of *Pf*Δ*mei2* (*n* = 7 mice) and WT *Pf*NF54 (*n* = 4 mice). Significance values (unpaired Mann–Whitney test): **p < 0.001. Dotted line: the cutoff as used in controlled human malaria infection (CHMI) at OHSU of 5 parasites/ml, assuming 7400 18 S copies/per parasite. **c** Analysis of blood samples from FRG huHep mice for presence of blood-stage parasites by in vitro cultivation of blood stages. Cultures, maintained in a semi-automated shaker system, were monitored for blood-stages for 28 days by microscopy analysis of Giemsa-stained thin and thick blood smears (see **d**) and by 18 S qPCR. Significance values (unpaired Mann–Whitney test): **p < 0.001. Dotted line: the 10 parasites/ml cutoff used in CHMI at LUMC, assuming 4252 18 S copies per parasite. **d** Blood-stage parasites in cultured blood samples collected from FRG huHep mice (m) after infection with *Pf*Δmei2 and WT PfNF54 sporozoites. Samples from in vitro cultures were analyzed at different days (**d**) for blood-stage parasitemia by microscopy (% of infected RBC).

Next, the liver-stage development of *Pf*Δ*mei2* and WT *Pf*NF54 parasites was analyzed in liver-chimeric humanized mice (FRG huHep mice) engrafted with high proportions of human red blood cells (RBC), injected intravenously (see Fig. 3a for a schematic representation of these experiments). The use of FRG huHep mice to mimic in vivo hepatocyte infection with *P. falciparum* sporozoites through to the blood-stage of infection has been previously described^28,29^. Briefly, mice were infected with 1 × 10(6) sporozoites of WT *Pf*NF54 or *Pf*Δ*mei2* followed by injection of human red blood cells at days 5 and 6 p.i. Blood was collected for qRT-PCR analysis at days 7 and 9 p.i., and mice were euthanized at day 9 p.i. for blood collection and cryopreservation. In WT *Pf*NF54-infected mice an average of 1,4 × 10(10) 18 S copies per ml was detected at day 7 (range: 1,1 × 10(10) to 2,0 × 10(10)) which increased on day 9 to an average of 1,2 × 10(11) copies (range 7,0 × 10(10) to 1,6 × 10(11)). In the *Pf*Δ*mei2*-infected mice the average number of 18 S copies was much lower at day 7 (2,4 × 10(6); range 1 × 10(6) to 4,9 × 10(6)) and dropped 10-fold at day 9 in 6 mice (3,2 × 10(5); range 3,7 × 10(4) to 1,1 × 10(6)) while in one mouse the number of 18 S copies remained similar (5,8 × 10(6) at day 7 and 7,3 × 10(6) at day 9. (Fig. 3b). Combined these observations show the presence of replicating parasites in WT *Pf*NF54-infected mice whereas in the *Pf*Δ*mei2*-infected mice replicating blood stages are absent. To determine if the WT *Pf*NF54 or *Pf*Δ*mei2* infected mice contained viable blood stage parasites, the cryopreserved blood of the FRG huHep mice collected at day 9 was cultured using standard in vitro culture conditions for blood-stage parasites. Samples from all WT *Pf*NF54-infected mice showed a >0.1% parasitemia after 5 days of culture. In contrast, none of the cultures of blood samples of the *Pf*Δ*mei2*-infected mice became blood-stage positive up to 28 days, as determined by microscopy of Giemsa stained smears and 18 S qRT-PCR analyses (Fig. 3c, d). The absence of parasites in the cultures of *Pf*Δ*mei2*-infected mouse blood suggests that the low number of 18 S copies detected at days 7 and 9 in these mice most likely results from the presence of dead parasite material released from *Pf*Δ*mei2-*infected hepatocytes in the blood. Overall, our results show that *Pf*Δ*mei2* sporozoites can effectively infect human hepatocytes in vitro, develop into large, replicating liver-stages that express merozoite-specific antigens but these maturing liver-stages fail to produce human RBC-infective merozoites in the livers of liver/blood-humanized mice.

### *PfΔmei2* genotyping by whole-genome sequencing confirms correct deletion of *mei2* and indicates absence of major rearrangements or integration of heterologous DNA sequences

The promising results of late growth arrest of *Pf*Δ*mei2* liver-stages warranted further characterization of *Pf*Δ*mei2* parasites to obtain regulatory approval for clinical testing of this LA-GAP. Clinical evaluation involves safety testing, i.e., analysis of adverse events in humans resulting from injection of *Pf*Δ*mei2* sporozoites or resulting from possible break-through blood infections, similar to procedures used in safety evaluation of the GA1 *P. falciparum* GAP^10^. Thorough identity testing and characterization of the genetic makeup of the parasites, in particular a confirmation of absence of unwanted genetic alterations which may impact the virulence of *Pf*Δ*mei2* parasites, is crucial for regulatory approval of their clinical use. We therefore analyzed the presence of undesirable integration of plasmid DNA sequences that were used to generate *Pf*Δ*mei2* and the presence of other genome rearrangements that might result from the genetic modification or mutations that may affect gene expression or drug-sensitivity. Four different plasmids with heterologous DNA sequences were used to create *Pf*Δ*mei2*, specifically, (i) a donor DNA plasmid containing two 34 bp *frt* (GAAGTTCCTATACTTTCTAGAGAATAGGAACTTC) sequences; (ii) two sgRNA plasmids containing the heterologous selectable-marker genes *bsd*, h*dhfr* and y*fcu* flanked by 3′ and 5′-utr sequences of *P. falciparum*-specific genes, employed to delete *mei2* by double cross-over integration; (iii) a fourth plasmid, containing a yeast FLPe recombinase expression cassette, which was used to remove nearly all heterologous DNA from the donor DNA plasmid that is introduced into the genome for deletion of *mei2*, specifically the *bsd* selectable-marker cassette. These heterologous DNA sequences, located between the two *frt* sequences are excised in the presence of FLPe recombinase, leaving one *frt* sequence in the genome. This method of removal of heterologous DNA by FLPe recombinase is similar to that described for the generation of the GA1^10^ that also contains a single *frt* site.

**Fig. 4:**
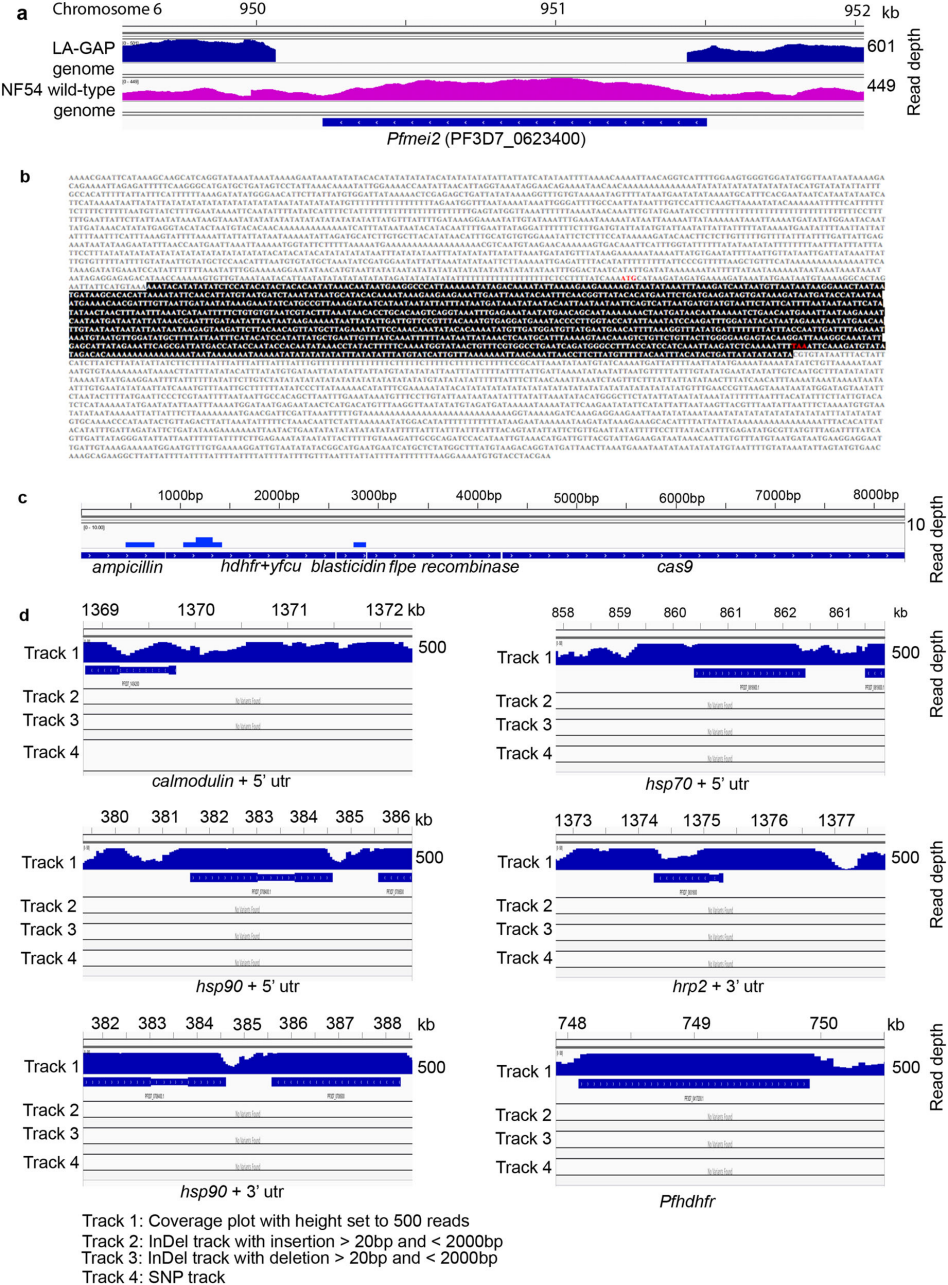
*Pf*Δ*mei2* genome sequence analysis. **a** The *mei2* locus of *Pf*Δ*mei2*. No sequence reads are mapped on the *mei2* coding sequence while reads map in the *mei2* up- and downstream regions. **b** Sequence of the *mei2* locus of *Pf*Δ*mei2*. The *mei2* flanking regions are unaltered and the expected *mei2* deletion event is shown by the preservation of the *mei2* targeting sequences (yellow). The *mei2* coding sequence (in red) is absent (start and stop codon in green). **c** Uniquely mapped sequence read (Illimina reads) coverage of heterologous sequences used in the DNA constructs/plasmids for generation of *Pf*Δ*mei2*. None of the sequences (*ampicilin*, h*dhfr-*y*fcu, blasticydin, flpe,* and *cas9*) were mapped with sequence reads from the *Pf*Δ*mei2* genome. **d** Read coverage, InDel, and SNP information of the following endogenous 5′- and 3′-*utr* sequences, used in DNA constructs/plasmids to drive gene expression. These sequences were unaltered in the *Pf*Δ*mei2* genome. The regions were: 5′UTR: *calmodulin*, PF3D7_1434200; *Hsp*70, PF3D7_0818900; *Hsp90*, PF3D7_0708400; 3′UTR: *Hpr2*, PF3D7_0831800; *Hsp90*, PF3D7_0708400; and the *dhfr/ts* locus, PF3D7_0417200.

First, diagnostic PCR analysis and Southern blot analyses of digested genomic DNA were used to confirm the correct deletion of *mei2* and subsequently the removal of the *bsd* selectable-marker cassette from the *mei2* locus in *Pf*Δ*mei2* by (Fig. 1b, c). In addition, PCR products that encompass the wild-type *mei2* and deleted DNA regions were cloned and sequenced, revealing that (i) the expected gene-targeting had occurred at the *mei2* locus resulting in deletion of *mei2*, ii) the *bsd* selection marker was absent in the *mei2* locus, and (iii) only a single *frt* sequence was present in the *mei2* locus (Fig. 1d, e). Next, the complete genome of *Pf*Δ*mei2* was sequenced and single nucleotide polymorphisms (SNPs), insertions and deletions were analyzed by comparison with the reference 3D7 *P. falciparum* genome, which is a cloned line of *Pf*NF54. This analysis showed that: (i) No reads were mapped on the *mei2* coding sequence, while they mapped to the *mei2* 3′ and 5′ *utr* sequences, confirming correct deletion of *mei2* and showing preservation of the flanking targeting sequences in the *Pf*Δ*mei2* genome (Fig. 4a, b); (ii) None of the following plasmid sequences are present in the *Pf*Δ*mei2* genome: *cas9*, *ampicillin*, the drug selection marker genes *bsd*, h*dhfr*, y*fcu* and the *flpe recombinase* gene (Fig. 4c); (iii) The endogenous 3′- and 5′*utr* sequences that were used in plasmids to drive gene expression were unaltered in the *Pf*Δ*mei2* genome, showing the absence of unwanted recombination events in these genomic regions (Fig. 4d). These sequences comprised 5′*utr* sequences of *calmodulin* (PF3D7_1434200), *Hsp70* (PF3D7_0818900), *Hsp90* (PF3D7_0708400) and 3′*utr* sequences of *Hpr2* (PF3D7_0831800); *Hsp86* (PF3D7_0708400) and *dhfr/ts* (PF3D7_0417200); iv) No detectable rearrangements in the *PfΔmei2* genome were observed by InDel analysis, using programs for identification of insertion and deletions; v) A total of 105 high-quality SNPs were identified in genes and only two of these 105 genes had a non-synonymous mutation: *eukaryotic translation initiation factor 2* (PF3D7_0107600, A > C position 3421, amino acid N114 to H) and a *Pfemp1* (PF3D7_0421100, A > T, position 914, amino acid Y350 to F). In conclusion, our analyses did not indicate the presence of unwanted integration of (heterologous) plasmid DNA sequences into the *Pf*Δ*mei2* genome or the presence of genome rearrangements, except for the deletion of *mei2,* that may impact other phenotypic/virulence characteristics of *Pf*Δ*mei2*.

### Drug sensitivity of *PfΔmei2* blood-stage parasites is comparable to that of WT *Pf*NF54 blood stages

**Fig. 5:**
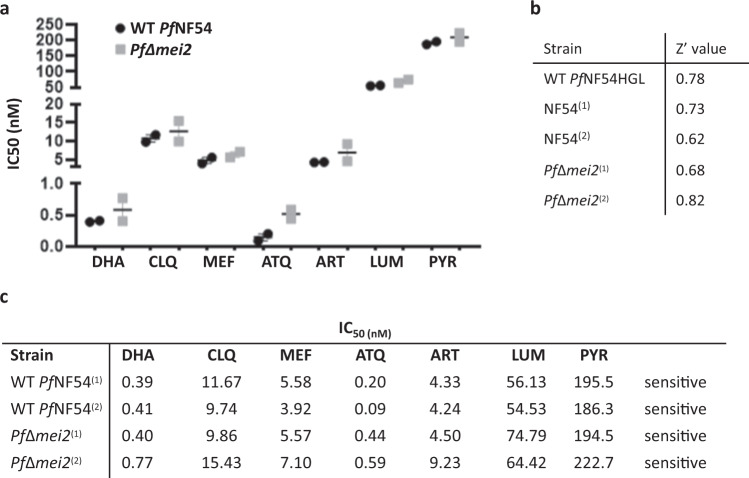
Sensitivity of *Pf*Δ*mei2* and WT *Pf*NF54 blood stages to seven antimalarial drugs. **a** Drug sensitivity of *PfΔmei2* (gray) and WT *Pf*NF54 (black) to seven antimalarial drugs was determined in an asexual Blood stage (ABS) SYBR Green drug assay. The graph shows the IC50 value with standard error of the mean (SEM) (see **c** below for the exact values). DHA dihydroartemisinin, CLQ chloroquine, MEF mefloquine, ATQ atovaquone, ART, artemisinin, LUM lumefantrine, PYR pyrimethamine. **b** Z’ values for each tested *Plasmodium falciparum* strain. The Table shows the Z’ values for each of the plates tested in the ABS replication assay. **c** IC50 values for each culture for the drugs shown in **a**. IC50 was determined using a four-parameter non-linear regression model using least-squares to find the best fit. Error bars represent the standard error of the mean.

An inherent safety feature of attenuated parasites is the ability to quickly cure any breakthrough blood infection with a variety of drugs. As noted above, the comparison of the genome sequences of *Pf*Δ*mei2* and WT 3D7 *P. falciparum* revealed the presence of a relatively low number of SNP’s. Non-silent mutations in coding and non-coding sequences may affect genes that encode proteins involved in sensitivity/resistance of blood-stage parasites to commonly used antimalarials. We therefore determined drug sensitivity of *Pf*Δ*mei2* and WT *Pf*NF54 blood-stages to seven commonly used drugs for treating (experimental) malaria infections (dihydroartemisinin, chloroquine, mefloquine, atovaquone, artemisinin, lumefantrine and pyrimethamine). Blood-stages of both parasite lines were sensitive to all drugs tested, with IC50 values in the nanomolar range (Fig. 5). These results indicate that SNPs detected in *PfΔmei2* did not impact on the sensitivity of *PfΔmei2* blood-stages to the drugs tested in this study.

## Discussion

To generate *P. falciparum* GAPs with a late liver-stage growth arrest, we selected four genes for deletion based on published phenotypes of rodent malaria parasite mutants, lacking the equivalent orthologous genes. In contrast to the rodent parasite data, three out of these four genes (*palm, hcs1, cbr*) appear to be essential for the formation of *P. falciparum* sporozoites inside the oocyst. A comparable discrepancy in gene function between rodent malaria parasites and *P. falciparum* has been reported for genes encoding apicoplast-located proteins involved in the transformation of Acetyl-CoA into Acyl-ACP for generation of fatty acids by the FASII pathway^18^. There is no proof that the three proteins selected, PALM, HCS1 and CBR, play a direct role in this (part of the) FASII-pathway, although CBR may operate in fatty acid elongation^30^ and the rodent *Plasmodium* PALM localizes to the apicoplast, which would be compatible with a function in the FASII pathway^20^. However, additional studies are required to unravel the biochemical pathways in which these proteins take an indispensable role for formation of *P. falciparum* sporozoites

In contrast to *palm, hcs1* and *cbr*, we found that *P. falciparum* GA2 lacking *mei2* phenocopies *P. yoelii*^24,25^ and *P. berghei mei2* gene-deletion mutants (this study). The rodent *mei2* knock-out malaria parasite mutants develop similarly to wild-type parasites in the blood, in the mosquito and during part of their intra-hepatic development process, but their growth is arrested during replication inside hepatocytes. Recently, a similar phenotype of late liver growth arrest has also been described for a *P. falciparum mei2* gene-deletion mutant by Goswami et al.^31^. We found a highly similar development of the *Pf*Δ*mei2* parasites into replication-competent, late liver-stages and the absence of formation of merozoites that infect human red blood cells. The MEI2 (or PlasMei2) protein is a member of a family of RNA-binding proteins containing an RNA recognition motif and is only expressed in late-liver-stages where it has a granular cytoplasmic location^31^. Detailed observations on the development of *P. falciparum* liver-stages lacking the MEI2 protein showed aberrant formation of cytomeres, structures formed in maturing schizonts during the formation of the multiple daughter merozoites and impaired DNA replication and segregation^31^. Similar to what has been observed by Goswami et al.^31^, we provide evidence that these schizonts do not produce merozoites that are capable of infecting RBC. In standardized experiments using FRG huHep mice transfused with human RBC^28,29^, no evidence was found for development of blood-stage infections after injection of high numbers (1 × 10(6)) of *Pf*Δ*mei2* sporozoites. These observations indicate a complete late-liver stage growth arrest of *P. falciparum* parasites lacking the MEI2 protein. However, occasional break-through blood infections were found in mice injected with more than 2 × 10(5) sporozoites of both *P. yoelii*^28^ and *P. berghei mei2* gene-deletion mutants (this study). These occasional break-through blood infections of the rodent malaria mutants may be specific for the rodent malaria species which have a short period of full liver-stage development of only two days compared to a period of more than six days for *P. falciparum*. However, in the mouse studies with the rodent malaria parasites larger groups of mice were infected with sporozoites of the *mei2* gene-deletion mutants compared to the number of FRG huHep mice injected with *PfΔmei2* sporozoites. In addition, infectivity of *P. falciparum* sporozoites may be lower in the FRG huHep mouse model than in humans and therefore formal proof of complete attenuation of *Pf*Δ*mei2*, parasites awaits controlled safety studies in humans^10^.

The phenotype of growth arrest in the liver of *Pf*Δ*mei2* is highly similar to the phenotype of *P. falciparum mei2* gene-deletion mutant (*P. falciparum* *mei2*^–^) reported by Goswami et al.^31^. Both mutants have been generated by CRISPR/Cas9-mediated gene deletion. However, differences exist in the plasmids used for deleting *mei2*, the deleted sequence and in drug-selection of the mutants. All phenotype analyses of the *P. falciparum* mei2^–^ were performed with a mutant that still contains the *hdhfr* selectable-marker cassette in the *mei2-* locus and no data is reported on whole-genome sequence analyses of the different *P. falciparum mei2*^*–*^ mutants with or without the *hdhfr* selectable marker. CRISPR/Cas9 modification can make off target gene mutations, not only at or near the target site, but also far from the target site^32,33^, mutations that may affect the phenotype of *P. falciparum* mutants, for example impacting virulence features or drug sensitivity.

To obtain regulatory approval for clinical evaluation of *Pf*Δ*mei2*, we performed detailed studies to investigate whether genetic modification procedures did not cause unwanted genetic alterations, possibly impacting the virulence of the genetically modified parasites. Our genotype analyses, including whole-genome sequencing, did not show unwanted integration of (heterologous) plasmid DNA sequences or genome rearrangements in *Pf*Δ*mei2* that may impact other phenotypic/virulence characteristics of *Pf*Δ*mei2*, except for the intentional deletion of *mei2* from the *Pf*Δ*mei2* genome. In addition, the sensitivity of *Pf*Δ*mei2* blood-stages to seven antimalarial drugs showed IC50 values in the nanomolar range similar to those found for the parent WT *Pf*NF54. Based on our observation, it can therefore be expected that the safety profile of growth-arrested *Pf*Δ*mei2* sporozoites in humans is highly similar to sporozoites of PfSPZ-GA1^10^, *Pf*SPZ Vaccine^1^ or WT *Pf*NF54 sporozoites administered under chloroquine prophylaxis^34^.

The results of the preclinical evaluation reported in this study have led to the regulatory approval of *Pf*Δ*mei2* (named GA2) for use in human studies by the Gene Therapy Office of the Dutch Ministry of Infrastructure and the Environment in the Netherlands, licensed to the Leiden University Medical Centre (GGO IM-MV 20-018_000) and subsequent approval of the Central Committee on Research Involving Human Subjects (CCMO; NL75577.000.21). Safety and subsequent efficacy studies using the CHMI model have currently been initiated to provide the answer to the key question of whether attenuated parasites with a growth arrest late in the liver substantially increase potency compared to Wsp vaccines consisting of attenuated parasites with an early-growth arrest, similar to what has been found in rodent studies^17^ and is expected based on studies using chemo-attenuated PfSPZ immunization^16^. Moreover, parallel assessment of immunological samples obtained from individuals exposed to EA-GAP (GA1 and PfSPZ Vaccine) or LA-GAP GA2 will provide unprecedented insight into the immunology of the liver stage of human malaria, thus far a black box. Most importantly, the prospect of a highly efficacious malaria vaccine using cutting-edge molecular technology provides hope for regaining control for malaria control programs struggling with resistance.

## Methods

### Experimental animals (ethics statement): Leiden, LUMC (The Netherlands)

Animal experiments were granted with a license DEC12042 and 14207 by Competent Authority after an advice on the ethical evaluation by the Animal Experiments Committee Leiden and were performed in accordance with the Experiments on Animals Act (Wod, 2014), the applicable legislation in the Netherlands in accordance with the European guidelines (EU directive no. 2010/63/EU). Experiments were executed in a licensed establishment for experimental animals. Mice were housed in ventilated cages with autoclaved aspen woodchip, fun tunnel, wood chew block and nestlets (12:12 hour (h) light-dark cycle; 21 ± 2 °C; relative humidity of 55 ± 10%) and fed with a commercially-prepared autoclaved, dry rodent diet pellets and provided with water, both available *ad libitum*. Female OF1 and C57BL/6 mice (6–7 weeks; Charles River Laboratories, France) were used. Experiments involving generation of mutant parasite lines and phenotype analyses were performed using highly standardized and approved protocols that have been developed to reduce the number of animals and minimize suffering and distress. Mice were killed (cardiac puncture under isoflurane anesthesia or CO_2_) at a parasitemia of 2–5%, before malaria-associated symptoms occur. Humane endpoints: the animals/body condition was thoroughly examined daily. Animals are humanely sacrificed in case the following defined end points are reached: visible pain (abnormal posture and/or movement), abnormal behavior (isolation, abnormal reaction to stimuli, no food and water intake). If distress of the animals is observed by the animal caretakers, this will be reported to the investigators and according to the aforementioned criteria, the animals will be taken out of the experiment and euthanized. In all experiments no mice were euthanized before termination of the experiment and no mice died before meeting criteria for euthanasia.

#### Mosquitoes

Mosquitoes from a colony of *Anopheles stephensi* (line Nijmegen SDA500) were used. Larval stages were reared in water trays (at 28 ± 1 °C; relative humidity 80%). Adult females were transferred to incubators at 26 ± 0.2 °C (relative humidity of 80%) and were fed with 5% filter-sterilized glucose solution. For the transmission experiments, 3 to 5 day-old mosquitoes were used. Following infection, the *P. berghei* and *P. falciparum*, infected mosquitoes were maintained at 21 °C and 26 °C, respectively, at 80% relative humidity.

#### Parasites

For generation of the rodent malaria LA-GAP *Pb*Δ*mei2*, the *P. berghei* ANKA reference line 1868cl1 was used (line RMgm-1320; www.pberghei.eu) which contains the reporter genes *mCherry* and *luciferase* under control of the constitutive *hsp70* and *eef1α* promoters, respectively, integrated into the neutral *230p* gene locus (PBANKA_0306000). This line does not contain a drug-selectable marker. Production of *Pb*Δ*mei2* and characterization of these parasites throughout their life cycle, including mosquito transmission, was performed under GMO permits IG 17-230_II-k en IG 17-135_III.

*P*. *falciparum* parasites NF54 strain^35^ was used as wild-type *P*. *falciparum* parasites *(*WT *Pf*NF54). Parasites from the *Pf*NF54 strain, and its derivative *Pf*3D7, are the most commonly used *P. falciparum* parasites in laboratory studies and in Controlled Human Malaria infections (CHMI;^36^). The complete genome sequences of *Pf*3D7 and *Pf*NF54 have been published^37,38^. The parasites of *Pf*NF54 and *Pf*3D7 have been deposited with the Malaria Research and Reference Reagent Resource Center (MR4; MRA-1000 and MRA-102), which was developed by the National Institute of Allergy and Infectious Diseases (NIAID) and is managed by the American Type Culture Collection (ATCC) (BEI Resources; https://www.beiresources.org/About/MR4.aspx). Parent parasites used for the generation of the *Pf*Δ*mei2* and the other gene-deletion mutants were obtained from a characterized good manufacturing process (GMP) produced working cell bank of the WT *Pf*NF54^35^, produced by Sanaria Inc^39,40^. WT *Pf*NF54 is sensitive to the following antimalarial drugs: atovaquone/proguanil, arthemeter/lumefantrine and chloroquine^41^.

For cultivation of *P*. *falciparum* blood-stage parasites^42^, Fresh human serum and human red blood cells (RBC) were obtained from the Dutch National Blood Bank (Sanquin Amsterdam, the Netherlands; permission granted from donors for the use of blood products for malaria research and microbiology tested for safety). Production of genetically modified parasites and characterization of these parasites throughout their life cycle, including mosquito transmission, was performed under GMO permits IG 17-134_II-k en IG 17-135_III.

#### Generation and genotyping of *P. berghei PbΔmei2*

The *P. berghei mei2* (PBANKA_1122300) gene was deleted by standard methods of transfection^43^ using a gene-deletion plasmid (PbGEM-300555, pL2206) obtained from PlasmoGEM (Wellcome Trust Sanger Institute, UK; http://plasmogem.sanger.ac.uk)^44^. This construct is designed to replace the *mei2* open reading frame (*orf*) by the h*dhfr::*y*fcu* selectable marker (SM) cassette by double cross-over homologous recombination. The SM cassette contains the h*dhfr::yfcu* flanked by the *P. berghei eef1α* promoter region and 3′ terminal sequence of pb*dhfr*. Before transfection, the construct was linearized by digesting with *Not*I. Parasites of line 1868cl1 were transfected with construct pL2206 (exp. 2834) and transformed parasites selected by positive selection with pyrimethamine^43^. Selected parasites were cloned by limiting dilution and cloned lines 2834cl1 and 2834cl2 were used for genotype analysis. Line 2834cl2 was further used to generate the gene-deletion mutant which is SM free. To remove the h*dhfr::*y*fcu* SM cassette from the genome of 2834cl2, the parasites were selected (negative selection) by treatment of infected mice with 5-fluorocytosine (5-FC)^45^. This treatment selects for parasites that have undergone homologous recombination between the two 3′-UTR of pb*dhfr* untranslated regions present in the integrated construct pL2206, flanking the h*dhfr::*y*fcu* cassette and thereby removing the SM^46^. Selection and cloning of the parasites resulted in the SM-free gene-deletion line *Pb*Δ*mei2* (2834cl2m1cl1). Correct integration of the construct and deletion of the *mei2* gene were confirmed by Southern blot analyses of Pulsed Field Gel (PFG)-separated chromosomes and diagnostic PCR analysis^43^. To show integration of the PlasmoGem construct containing the h*dhfr::*y*fcu* SM or removal of the h*dhfr::*y*fcu* SM by negative selection, the PFG-separated chromosomes were hybridized with a mixture of two probes: a probe recognizing the h*dhfr* gene and a control probe recognizing gene PBANKA_0508000 on chromosome 5^47^. PCR primers for genotyping are listed in Supplementary Table 1.

### Phenotyping of *P. berghei PbΔmei2*

The in vivo multiplication rate of asexual blood stages was determined during the cloning procedure of the different QC mutants^48^. Infection of ‘, For collection and counting of sporozoites from infected *An. stephensi* mosquitoes^49^ mosquito salivary glands were manually dissected (21 days after feeding). Salivary glands were collected in RPMI medium, homogenized and filtered (40 µm Falcon, Corning, NL). Free sporozoites were counted in a Bürker counting chamber using phase-contrast microscopy. The human-hepatoma cell line Huh7^50^ was used for in vitro cultivation of liver-stages. Briefly, 5 × 10(4) isolated sporozoites were added to monolayers of Huh7 cells on coverslips in 24 well plates (with confluency of 80–90%) in complete RPMI-1640 medium supplemented with 10% (vol/vol) fetal bovine serum, 2% (vol/vol) penicillin-streptomycin, 1% (vol/vol) GlutaMAX (Invitrogen), and maintained at 37 °C with 5% CO_2_. At 24, 48 and 72 hours post infection (p.i.) nuclei were stained with Hoechst-33342 at a final concentration of 10 µM and live imaging of mCherry-expressing parasites was performed using a Leica fluorescence MDR microscope (×40 magnification). Pictures were recorded with a DC500 digital camera microscope using Leica LAS X software with the following exposure times: mCherry: 0.7 s and Hoechst 0.136 s (1× gain). Liver-stage parasite sizes were measured using Leica LAS X software by determining the area of the parasite at its greatest circumference using the mCherry-positive area (µm^2^).

To determine the attenuation phenotype of *Pb*Δ*mei2*, C57BL/6 mice were infected with 5 × 10(3), 5 × 10(4), or 2 × 10(5) sporozoites of WT or *Pb*Δ*mei2*. Isolated sporozoites, suspended in RPMI-1640 medium, were intravenously injected into the tail vein (200 μl per mouse). Parasite liver loads in live mice were quantified by real-time in vivo imaging^51^. Parasite liver loads were visualized and quantified by measuring luciferase activity of parasites in whole bodies of mice at 44, 56, and 65 h p.i using the IVIS Lumina II Imaging System (Perkin Elmer Life Sciences, Waltham, USA). D-luciferin was dissolved in PBS (100 mg/kg; Caliper Life Sciences, USA) and 60 µl injected subcutaneously in the neck. Measurements were performed within 8 min after the injection of D-luciferin. Quantitative analysis of the bioluminescence of whole bodies was performed by measuring the luminescence signal intensity (RLU; relative light units) using the ROI (region of interest) settings of the Living Image® 4.5.5 software. Mice were monitored for blood-stage infections by Giemsa-stained blood smears made at day 4 to 30 p.i. The prepatent period (measured in days after sporozoite challenge) is defined as the day when a blood-stage infection with a parasitemia of 0.5–2% is observed^47^.

#### Generation and genotyping of four *P. falciparum* gene-deletion mutants (see Supplementary Table 1 for primer sequences)

*PfΔpalm:* The *palm* gene (PF3D7_0602300) was deleted using a CRISPR/Cas9 approach where first a plasmid was introduced into parasites as an episome containing the *cas9* gene^52^, CRISPR/Cas9 system in Plasmodium falciparum using the centromere plasmid^53^. This plasmid, pLf0086, contains the h*dhfr* SM (with the *P. chabaudi dihydrofolate reductase thymidylate synthase* gene promoter (*Pc*DT; PCHAS_0728300) and the *cas9* gene (with the *P. falciparum heat shock protein 90* gene promoter the (PF3D7_0708400; *Pfhsp90*). To generate pLf0086, plasmid pLf0070 (pDC2-cam-Cas9-U6.2-hdhfr)^54,55^ was digested with *BamH*I to remove the sgRNA/U6 cassette. Re-circularized plasmid in the *BamH*I site was termed pLf0086. Transfection of WT *Pf*NF54 parasites with plasmid pLf0086 was performed by the method of spontaneous plasmid uptake from plasmid-loaded RBC^56^. Transfected parasites were selected by treatment with the drug WR99210 (2.6 nM) for a period of two weeks (until parasites were detectable in Giemsa-stained thin blood films) to select for parasites containing the plasmid pLf0086 episomally (Exp.121). Subsequently, selected parasites were simultaneously transfected with two sgRNA/donor DNA plasmids, pLf0124, pLf0125. Both plasmids contain two homology regions (HR) targeting *palm*, a *blasticidin-S-deaminase* (*bsd*) SM cassette (with the *P. falciparum hsp70* promoter; *Pfhsp70;* PF3D7_0818900) and a *palm* sgRNA cassette. Each plasmid contains a different sgRNA. To generate the *palm* targeting vectors, a basic plasmid pLf0103 was designed that contains the *bsd* SM cassette. To generate the *bsd* SM cassette, a gBlock was designed and ordered (https://eu.idtdna.com/pages), containing the *bsd* gene flanked by two 34 bp f*lippase recognition target* (*frt*) sequences (GAAGTTCCTATTCTCTAGAAAGTATAGGAACTTC;^57^) (Supplementary Fig. 7). These sequences allow to recycle the SM cassette^57^. This fragment was cloned into the *P. berghei* transfection construct pL0034 (RMgm-687; www.pberghei.eu^46^;) using the restriction enzymes *EcoR*I/*Hind*III resulting in intermediate plasmid SKK159. The *pfhsp70* promoter was obtained by PCR amplification (KOD Hot Start DNA Polymerase, Merck Millipore) using primers p1/p2 and cloned into the intermediate plasmid SKK159 using the restriction enzymes *Kpn*I/*Xho*I resulting in the intermediate plasmid SKK160. Finally, the 3′*utr* of *P. falciparum histidine rich protein 2 (Pfhrp2;* PF3D7_0831800) was obtained by PCR amplification (KOD Hot Start DNA Polymerase, Merck Millipore) using primers p3/p4 and cloned into SKK160 using the restriction enzymes *Not*I/*Avr*II resulting in the final basic plasmid pLf0103. This construct contains additional restriction sites for introducing homology/targeting sequences to target any gene of interest, such as *Nae*I*/Sac*II and *Apa*I*/Hind*III and for introducing the sgRNA/U6 cassettes, such as *Aat*II*/ BamH*I (see below). Plasmid pLf0039 with the *P. falciparum u6* RNA promoter (PF3D7_1341100) containing the *BtgZ*I adaptor^58^ was used two generate two sgRNA expression cassettes for two intermediate plasmids containing sgRNA009 (pLf0110) and sgRNA010 (pLf0111). The guide sgRNA sequences for *palm* (sgRNA009 and sgRNA010) were identified using the Protospacer software (alpha version; https://sourceforge.net/projects/protospacerwb/files/Release/) and were amplified using the primers p5/p6 and p7/p8. These sgRNAs were selected based on the best off target hits score throughout the genome given by Protospacer and the total number of mismatches of the sgRNA with respect to the Protospacer adjacent motif site. Two 20 bp primer guide sgRNAs, surrounded by 15 bp vector-specific DNA necessary for InFusion cloning (HD Cloning Kit; Clontech), were annealed and used to replace the *BtgZ*I adaptor^58^, resulting in intermediate plasmids pLf0100, pLf0101, that were digested with *Bln*I/ *Nru*I for evaluation successful cloning and confirmed by Sanger sequencing using primers p9/p10. These constructs contain additional restriction sites for lifting the complete *u6* cassette including the sgRNA, such as *Aat*II*/BamH*I (see below). Next, two different sgRNA/donor DNA constructs, containing each of the sgRNA as well as the donor DNA sequences, were generated in multiple cloning steps resulting in pLf0124 and pLf0125. These constructs contain both the sgRNA expression cassettes and the *bsd* SM cassette. To generate the *palm* targeting vectors, plasmid pLf0103 was modified by introducing two HRs, HR1 and HR2, targeting *palm*. HR1 was amplified using primers P11/12 and HR2 with p13/p14 from WT *Pf*NF54 genomic DNA. HR2 was cloned into pLf0103 using restriction sites *Apa*I*/Hind*III, resulting in intermediate plasmid F171. Subsequently, HR1 was cloned into F171 using *Nae*I*/Sac*II, resulting in intermediate plasmid pLf0110 (F177). These plasmids are used to introduce sgRNA/U6 cassette from the intermediate plasmids pLf0100 and pLf0101, containing sgRNA009 and sgRNA010 respectively (using restriction sites *Aat*II*/ BamH*I) to generate pLf0124 and pLf0125, respectively.

Parasites of Exp.121 were transfected with the two plasmids pLf0124 and pLf0125 (a mixture of 50 μg of each circular plasmid in 200 μl cytomix) using standard transfection methods^59^. Selection of transfected parasites was performed by applying double-positive drug pressure from day 3 until day 9 after transfection using the drugs WR99210 (2.6 nM) and Blasticidin (BSD, 5 µg/ml). On day 9 drug pressure was removed and parasites were maintained in drug-free medium until parasites were detectable in thin blood-smears (day 15 after transfection). Selected parasites were then grown without both drugs until the parasitemia reached over 10%, followed by a second BSD selection (5 µg/ml) for a period of 7 days, resulting in parasite population Exp.167 (*PfΔpalm-1*) and Exp.169 (*PfΔpalm-2*). After drug selection, diagnostic PCR^42^ was performed from material isolated from iRBC. Correct replacement of the *palm* gene with the *bsd* cassette in the parasites after the second BSD selection in *PfΔpalm-1* and *PfΔpalm-2* parasites was confirmed by long-range PCR amplification (LR-PCR) (primers P15/P16) and standard PCR amplification of the *palm* open reading frame (primers p17/p18) and the *bsd* SM cassette (primers p19/p20). The PCR fragments were amplified using KOD Hot Start Polymerase (Merck Millipore) following standard conditions with annealing temperatures of 50.5 and 51 °C for 25 s and an elongation step of 68 °C for 3 min.

*Pf*Δ*cbr*: The *Pfcbr* gene (PF3D7_1367500) was deleted in WT *Pf*NF54 parasites by standard methods of CRISPR/Cas9 transfection^58,59^ using a sgRNA-expressing plasmid pLf0178, containing the *cas9* expression cassette, guide-RNA expression cassette and an h*dhfr* SM cassette, in combination with a donor DNA plasmid pLf0179 that contains a *bsd* SM cassette (linked to *gfp*, and separated with skip peptide 2 A; *bsd*-2A-*gfp*) for positive selection and a y*fcu* SM cassette for negative selection. The sgRNA-expressing plasmid was generated as follows: pLf0070^54^ was digested with *Bbs*I and the sgRNA067 was selected using the CHOPCHOP webtool (https://chopchop.cbu.uib.no/)^60^ and subsequently cloned into the pLf0070 using primers p21/p22. In brief, the primers (100 µM each primer) were phosphorylated with T4 polynucleotide kinase (10 Units per reaction) during 30 min at 37 °C, followed by an annealing program of 5 min incubation at 94 °C and a ramp down to 25 °C at 5 °C per min, and subsequently ligated into the *Bbs*I digested pLf0070 vector using T4 ligase (5 units) resulting in the plasmid pLf0178.

The donor DNA plasmid pLf0179 was generated to replace the *Pfcbr* open reading frame with the *bsd* SM cassette linked to *gfp* (*bsd*-2A-gfp). For generation of pLf0179, the HR1 and HR2 regions of *Pfcbr* were amplified from WT *Pf*NF54 genomic DNA using primers p23/p24 and p25/p26. Fragments were digested with *Hind*III/*Apa*I and *Nhe*I/*BamH*I respectively and ligated into plasmid pLf0169 to obtain pLf0179. Plasmid pLf0169 was generated as follows: a new gBlock was designed and ordered (https://eu.idtdna.com/pages), containing the *bsd-*2A-*gfp* genes flanked by the two *frt* sequences. This fragment was cloned into the intermediate construct pL0f103 (see above) to replace the *bsd* cassette by *bsd*-2A-*gfp* using the restriction sites *Bsab*I/*Avr*II, resulting in the intermediate plasmid pLf0165. A second intermediate plasmid F213 was created amplifying the complete y*fcu* SM cassette controlled by the *pfhsp90* promoter and the 3′ terminator sequence from *P. berghei dihydrofolate reductase thymidylate synthase* gene (Pb*dhfr/ts;* PBANKA_0719300*;* 3′*PcDT*, PCHAS_0728300), from the existing vector pLf0003 (pHHT-FRT-Pf36^57^) using the primers p27/p28. The complete cassette was cloned into the vector pJET1.2/blunt (thermo scientific) using the restriction enzyme *EcoR*V. Finally the y*fcu* SM cassete from the F213 plasmid was subcloned into the vector pLf0165 using the restriction sites *Stu*I for the pLf0165 and *Pvu*II/*Stu*I for the *yfcu* SM, resulting in the plasmid pLf0169.

Transfection of WT *Pf*NF54 parasites with plasmids pLf0178 and pLf0179 was performed by spontaneous plasmid uptake from plasmid-loaded red blood cells cultured^59^. Transgenic parasites were selected by applying ‘double’ positive selection 72 h after transfection with the drugs WR99210 (2.6 nM) and BSD (5 µg/ml) during 7 days. Subsequently, both drugs were removed from the cultures until thin blood-smears were parasite-positive, followed by applying negative selection by addition of 5-FC (1 µM) in order to eliminate parasites that retained the Donor DNA construct as episomal plasmid. Negative drug pressure in the cultures was maintained until thin blood-smears were parasite-positive. After negative selection parasites (Exp. 236) were harvested for genotyping by diagnostic PCR and Southern analysis^58,59^. To confirm the integration and the presence of the *bsd-*2A-*gfp* cassette, 5′-integration, *3*′ integration, and *bsd*, PCRs were performed using the primers p29/p30, p31/p32 and p20/p33 respectively. In addition the absence of the *pfcbr* open reading frame was confirmed using the primers p34/p35. The PCR fragments were amplified using KOD Hot Start Polymerase (Merck Millipore) following standard conditions with annealing temperatures of 50, 55, 60 °C for 10 s and an elongation step of 68 °C. Southern blot analysis was performed with gDNA digested with *EcoR*I and *Nco*I (4 h at 37 °C) in order to confirm the deletion of *Pfcbr*. Digested DNA was hybridized with a probe targeting the *Pfcbr HR2*, amplified from WT *Pf*NF54 genomic DNA by PCR using primers p25/p26, and the ampicillin probe (*amp*), was amplified using primers p36/p37.

*Pf*Δ*hcs1*: The *Pfhcs1* gene (PF3D7_1026900) was deleted in WT *Pf*NF54 parasites by standard methods of CRISPR/Cas9 transfection^58,59^ using two different sgRNA-expressing plasmids, containing the *cas9* expression cassette, guide-RNA expression cassettes, and h*dhfr* SM cassette, in combination with donor DNA plasmid pLf0191 that contains a *bsd*-2A-*gfp* SM cassette. The two different sgRNA-expressing plasmids were generated as follows: pLf0070^54^ was digested with *Bbs*I and sgRNA074 and sgRNA075 (selected with CHOP-CHOP webtool) were cloned using primers p38/p39 and p40/p41, respectively, resulting in the plasmids pLf0193 and pLf0194. The donor DNA plasmid pLf0191 was generated to replace the *Pfhcs1 open reading frame* with a *bsd*-2A-*gfp*SM cassette flanked by two *frt* sequences. The HR1 and HR2 targeting regions of *Pfhcs1* were amplified from WT PfNF54 genomic DNA using the primers P42/P43 and P44/P45 respectively (Supplementary Table 1). Fragments were digested with *Hind*III/*Asc*I and *Sac*II/*Nhe*I and ligated into plasmid pLf0169 to obtain pLf0191. Transfection of WT *Pf*NF54 parasites with constructs pLf0191, pLf0193, and pLf0194 was performed by spontaneous plasmid uptake from plasmid-loaded red blood cells cultured^59^ and selection of *PfΔhcs1* parasites was as described above for generation of *PfΔcbr*, resulting in the line *Pf*Δ*hcs1* (Exp. 252). For genotyping *Pf*Δ*hcs1* parasites, diagnostic PCR was performed. To confirm the integration and the presence of the *bsd-*2A-*gfp* cassette, 5′-integration, 3′ integration PCRs were performed using the primers p46/p47, p48/p49 respectively, additionally the absence of the *pfhcs1* open reading frame was confirmed using the primers p50/p51. The PCR fragments were amplified using Phusion DNA Polymerase (NEB) following standard conditions with annealing temperatures of 58 °C for 30 s and an elongation step of 68 °C.

*Pf*Δ*mei2*: The *mei2* gene (PF3D7_0623400) was deleted in WT *Pf*NF54 parasites by standard methods of CRISPR/Cas9 transfection^58^ using a donor DNA plasmid pLf0105 and two different sgRNA-donor containing plasmids, pLf0080 and pLf0092, targeting the *mei2* gene.

For generation of plasmid pLf0105, two homology regions targeting the *mei2* gene were introduced in pLf0103. HR1 was amplified from WT *Pf*NF54 genomic DNA using primers P52/P53 and HR2 with primers P54/P55. The PCR fragments were sequenced after TOPO TA (Invitrogen) subcloning and subsequently cloned into pLf0103 using restriction sites *Nae*I/*SacI*I and *Apa*I/*Hind*III, resulting in plasmid pLf0105. To generate the two sgRNA-donor-containing vectors, plasmid pLf0070^54^ was digested with *Bbs*I and sgRNA030 and sgRNA032 were cloned, as described above, using primers P56/P57 and P58/P59, respectively, resulting in the plasmids pLf0080 and pLf0092. Transfection of WT *Pf*NF54 (Leiden vial 9002 obtained from Nijmegen; NF54 54/329 4514) with constructs pLf0105, pLf0080 and pLf0092was performed by spontaneous plasmid uptake from plasmid-loaded RBC^59^. Selection of transfected WT *Pf*NF54 parasites was performed by applying double positive selection (as described for selecting for generation of *PfΔcbr)* for a period of 6-19 days. After this treatment period with two drugs, cultures were maintained in drug-free medium until parasites were detectable in Giemsa-stained thin blood smears (a period of three weeks). Subsequently, parasites were treated for one week with BSD (5 μg/ml), resulting in parasite population Exp.151 (*Pf*Δ*mei2*a parasites; Fig. 1). Subsequently, selected parasites were cloned by limiting dilution. In order to remove the *bsd* SM cassette from the genome of *Pf*Δ*mei2*a, blood stage parasites of the uncloned population of *Pf*Δ*mei2*-a (Exp. 151) were transfected with plasmid pLf0120 that contains a *flpe* recombinase expression cassette (Fig. 1). To create plasmid pLf0120, we used plasmid pMV-FLPe^57^ (pLf0038) that contains a *bsd* SM cassette and an *flpe* recombinase expression cassette. We first replaced the *bsd* SM cassette of plasmid pMV-FLPe with the h*dhfr-yfcu* SM cassette of plasmid pLf0039^58^ (*Hind*III/*Kpn*I) to create pLf0120. The h*dhfr-*y*fcu* gene is flanked by the promoter of the *Pfhsp86* and the Pb*dhfr/ts* short (0,5 kb) terminator sequences. The *flpe* gene is flanked by the promoter of the *Pfhsp90* from *Pf*Dd2 strain (PfDd2_070012600;) and Pb*dhfr/ts* long (1 kb) terminator sequences. After transfection of *Pf*Δ*mei2*a blood stages with pLf0120 (as described above), cultures were treated for a period of six days (day 3–9) with WR99210 (2.6 nM), followed by a period of 2 weeks culture without WR99210 treatment. Subsequently, selected parasites were cloned by limiting dilution. DNA from iRBC was obtained from 10 ml cultures (parasitemia 3–10%). Diagnostic PCR was performed using primer pair (P60/P61). As a control, the gene *p47* (PF3D7_1346800) was PCR amplified using primers 8428/8756 (P62/P63). Southern blot analysis was performed with gDNA digested with *Xmn*I (4 h at 37 °C) in order to confirm the deletion of *Pfmei2*. Digested DNA was hybridized with probes targeting the *Pfmei2* (a fragment of 539 bp of the *mei2* coding sequence amplified using primers P64/P65, Pf*p47* gene (PF3D7_1346800, as a control fragment of 3910 bp, amplified using primers P62/P66), *ampicillin* gene (amp probe; amplified using primers P36/P37), the *bsd* SM (amplified using primers P67/P20), the *cas9* (amplified using primers P68/P69 and the *flpe* gene (amplified using primers P70/P71). In order to confirm the precise nature of the genetic deletion, a PCR product that encompass the *mei2* gene-deletion region of *Pf*Δ*mei2* was cloned and sequenced. This PCR product was obtained using primer pair P72/P73, cloned in pJET (Thermo Fisher Scientific) and sequenced using primers P74/P75.

### Phenotyping of *P. falciparum* mutants *PfΔpalm*, *PfΔhcs1* and *PfΔcbr*

The growth rate of asexual blood-stages (parasitemia) was monitored by determination of parasitemia in standard in vitro cultures (in a semi-automated shaker incubator system) for a period of 4 days with a starting parasitemia of 0.1%^42^. Parasitemia was determined by counting infected RBC in Giemsa-stained thin blood films in three independent experiments. Gametocyte production and exflagellation^54^ were quantified in gametocyte cultures.

For analysis of mosquito stages (oocysts and sporozoites) *An. stephensi* mosquitoes were infected with day 14 gametocyte cultures using the standard membrane feeding assay (SMFA)^59,61^. Oocysts and salivary gland sporozoites were counted at days 9 and day 21 p.i., respectively. For counting sporozoites, salivary glands from 10 to 15 mosquitoes were dissected, collected in 100 µl of PBS and homogenized using a grinder. Sporozoites were counted using a Bürker cell counter using phase-contrast microscopy.

Oocysts were analyzed in manually dissected midguts using a Leica MZ16 FA stereo-fluorescent microscope. The midguts were imaged with a Leica MZ camera at ×10 magnification using Leica LAS X software. Individual oocysts were observed under a Leica DM2500 light microscope and documented with at ×100 using Leica DC500 digital camera using Leica LAS X software. Sg-sporozoite numbers were analyzed in infected mosquitoes at day 18–21 p.i. For counting sporozoites, salivary glands from 30–0 mosquitoes were dissected and homogenized using a grinder in 100 μl of RPMI-1640 medium (pH 7.2) and sporozoites were analyzed in a Bürker cell counter using phase-contrast microscopy^54^.

#### Phenotyping of *P. falciparum* mutant *PfΔmei2*

The growth rate of asexual blood-stages and analysis of mosquito stages (oocysts and sporozoites) in *An. stephensi* mosquitoes were performed as described in the previous section for the other three gene-deletion mutants.

BioIVT hepatocytes: Analysis of the development of WT *Pf*NF54 and *Pf*Δ*mei2* parasites in primary human hepatocytes^58^ was performed as follows. Liver-stages of WT *Pf*NF54 and *Pf*Δ*mei2* were cultured in vitro using cryopreserved primary human hepatocytes obtained from BioIVT (Belgium) and thawed according to the instructions of the manufacturer. Cells were seeded at a density of 60,000 cells/well in a collagen-coated 96-well clear-bottomed black plate for 2 days. Medium was refreshed daily (hepatocyte medium: Williams’s E medium supplemented with 10% heat-inactivated fetal bovine serum, 2% penicillin-streptomycin, 1% fungizone, 0.1 lU/ml insulin, 1.6 μM dexamethasone). Per well, 7 × 10^4^ freshly dissected WT *Pf*NF54 *and PfΔmei2* sporozoites were added to the hepatocyte monolayer. After a quick spin (10 min at 1900 *g*), the plate was incubated at 37 °C under 5% CO_2_. The medium was replaced with fresh hepatocyte culture medium 3 h p.i., and daily for 9 days thereafter. At days 3, 5, 7, and 9 p.i., hepatocytes were fixed with 4% paraformaldehyde in 1× PBS for 30 min. After fixation the wells were washed three times with 1× PBS and permeabilized with 300 μl of 0.5% triton in 1× PBS during 1 h and then blocked with 10% of FCS in 1× PBS for 1 h. Fixed cells were washed with 1× PBS and standard IFA was performed using antibodies against (1) the cytoplasmic protein *Pf*HSP70 (PF3D7_0818900; rabbit anti-*Pf*HSP70-PE/ATTO 594 conjugated primary antibody; 1:200 dilution of 100 μg/ml stock solution StressMarq, Biosciences, NL); (2) the plasma membrane surface protein MSP1 (PF3D7_0930300; mouse monoclonal antibody 1:1000 of 4.0 mg/ml stock solution obtained from The European Malaria Reagent Repository, Edinburgh, UK) (3) and the parasitophorous membrane protein EXP1 (PF3D7_1121600, mouse monoclonal antibody (1:200 of 4.0 mg/ml stock solution obtained from The European Malaria Reagent Repository, Edinburgh, UK). In addition, cells were stained with Hoechst-33342 for nuclear staining. Cells were mounted in Image-iT signal Enhancer (Invitrogen Thermofisher, USA) and examined with a SP8 Leica confocal microscope at ×100 magnification. Number of infected hepatocytes, antibody staining, and size of parasites were analyzed using ImageJ.

LONZA hepatocytes: Cryopreserved primary human hepatocytes purchased from Lonza Bioscience were thawed and seeded in μ-Slide 18 Well ibiTreat coverslip (IBIDI, Gräfelfing, Germany), pre-coated with rat-tail collagen I (BD Bioscience, USA) in William’s E medium (Gibco) supplemented with 10% fetal clone III serum (FCS, Hyclone), 100 u/mL penicillin and 100 ug/mL streptomycin (Gibco), 5 × 10^−3^ g/L human insulin (Sigma-Merck), 5 × 10^−5^ M hydrocortisone (Upjohn Laboratories SERB, France) at 37 °C in 5% CO_2_. The next day, cells were overlaid with matrigel (Corning) and medium was then renewed every 2 days. Three days later, sporozoites were isolated by aseptic hand dissection of salivary glands of *PfΔmei2*- and WT PfNF54-infected mosquitoes. Matrigel was removed from hepatocyte culture and 30,000 sporozoites were inoculated to cells before centrifugation at 560×*g* for 10 min at RT and further incubation at 37 °C, 5% CO_2_. Three hours later, infected cultures were covered with matrigel prior to addition of fresh cell culture medium. Medium was renewed every day, until cell fixation at the chosen times. Infected cultures were fixed with 4% paraformaldehyde for 15 min at room temperature and liver-stage parasites were immunostained with polyclonal anti-PfHSP70 murine serum prepared in the lab, anti-PfEXP1 (kindly provided by Pr Jude Przyborski) and revealed with anti-mouse IgG Alexa Fluor® 594 and anti-rabbit IgG Alexa-Fluor 488-conjugated, respectively (Invitrogen). DAPI was added to visualize nuclei. Parasite number and size were determined using a Cell Insight High Content Screening platform equipped with the Studio HCS software (Thermo Fisher Scientific) at the CELIS platform (ICM, La Pitié-Salpêtière, Paris). Graphs and statistical analysis were done using Prism 8.4.3 software. The areas of parasites were compared using the Mann–Whitney *U* test.

Liver stage development of both *Pf*Δ*mei2* and WT *Pf*NF54 parasites were analyzed in liver-chimeric humanized mice (FRG huHep mice) purchased from Yecuris Corporation (Tualatin, OR) and housed at Oregon Health and Science University (OHSU) as per manufacturer’s recommendation. All studies were performed according to the regulations of the Institutional Animal Care and Use Committee (IACUC; protocol IP00002077). Female *An. stephensi* mosquitoes, aged 3–5 days, were infected with *PfΔmei2* and WT *Pf*NF54 at LUMC (Leiden, the Netherlands)^54,59^. 12 days after feeding, mosquitoes were shipped to OHSU. Sporozoites were isolated by salivary gland dissection from infected mosquitoes at day 16 p.i. at OHSU for infection of the FRG huHep mice. Isolated salivary gland sporozoite were run over glass wool to remove contaminating mosquito material and sporozoites enumerated by hemocytometer. FRG huHep mice (*Pf*NF54 WT *n* = 4; *Pf*Δ*mei2* *n* = 7) were infected by intravenous injection (retro-orbital injection) of 10(5) sporozoites in a 100 µl volume of phosphate buffered saline. Five days p.i., FRG huHep mice were injected intravenously with 400 µl freshly washed human type AB red blood cells (RBC; 70% hematocrit) supplemented with 0.035 mg/mouse clodronate (Formumax CloLip) and penicillin/streptomycin antibiotic (50 units penicillin, 50 µg streptomycin/ml, Sigma). The following day, FRG huHep mice were injected intraperitoneally with 700 µl freshly washed human type AB RBC (70% hematocrit). At 7 and 9 p.i. 100 µl of blood was collected into 2 ml NucliSens buffer (Biomerieux) for qRT-PCR analysis. At day 9 p.i. mice were euthanized and blood collected for cryopreservation in glycerolyte. To analyze the presence of blood stages in blood samples collected from the FRG huHep mice (day 7, 9) 18 S qRT-PCR^62^ quantification of blood-stage parasites was performed. In addition to the qRT-PCR analyses, the cryopreserved blood of all FRG huHep mice (collected at day 9) was used for in vitro cultivation of parasites, performed at the LUMC (Leiden, the Netherlands), to assess the presence/absence of blood stage parasites. For these experiments, a standard protocol for in vitro cultivation of blood stages was used^58,59^. Cultures, maintained in a semi-automated shaker system, were monitored for blood stage parasites for a period of 28 days by analyzing Giemsa stained thin and thick blood smears and 18 S qRT-PCR and fresh RBC (100 µl packed RBC in 1 ml culture medium) were added to the cultures weekly. Briefly, total DNA was isolated from blood samples using QIAamp DNA Blood Mini Kit (Qiagen, NL) in accordance with the manufacturer’s instructions, and a one-step qPCR Hotstar mastermix (Qiagen, NL) assay specific for the 18 S ribosomal subunit of *P. falciparum* was used to quantify the presence of parasites (forward primer: 5′-CCGACTAGGTGTTGGATGAAAGTGTTAA-3′; reverse primer: 5′-AACCCAAAGACTTTGATTTCTCATAA-3′; Probe: 5′-FAM- CTTTCGAGGTGACTTTTAGAT- MGB (quencher)^63^; cycling profile: 15 min at 95 °C followed by 45 cycles of 30 sec at 95 °C, 20 sec at 60 °C and 30 sec at 72 °C. The resulting CT values were compared to a standard curve of a known quantity of 18 S plasmid spanning 100 to 10(8) copies. To determine positive/negative parasite samples, the same cutoff as is used in controlled human malaria infection (CHMI) of 5 parasites/ml, assuming 7400 18 s copies/parasite (at OHSU) and 5 parasites/ml, assuming 4252 18 s copies/parasite.

Uncropped and unprocessed scans of gels and blots from (supplementary) Figs. are shown in Supplementary Figs. 8–13.

### Whole-genome sequencing of *PfΔmei2*

Genomic DNA of *PfΔmei2* was isolated from blood stage-infected RBC obtained from 10 ml cultures (parasitemia 3–10%). RBC were pelleted by centrifugation (1150 g; 5 min.) and subsequently lysed with 5–10 ml of cold (4 °C) erythrocyte lysis buffer (10× stock solution 1.5 M NH4Cl, 0.1 M KHCO3, 0.01 M Na2EDTA; pH 7.4). Pelleted parasites were treated with RNAse and proteinase-K before genomic DNA isolation by standard phenol-chloroform methods. Whole-genome sequencing was performed at the King Abdullah University of Science and Technology (KAUST, Thuwal, Saudi Arabia Prof. Arnab Pain). Genomic DNA quantification was carried out using Qubit-based colorimetric assay (Invitrogen). A total of 200 ng of DNA was used for DNA library preparation using NebNext Ultra II DNA library prep kit for Illumina (NEB). Upon library quantification and size verification, DNA library sequencing was carried out on a MiSeq platform (Illumina) that produced 2 × 150 bp paired-end reads. The quality of the raw reads was assessed using FATSQC (http://www.bioinformatics.babraham.ac.uk/projects/fastqc). Low-quality reads and Illumina adaptors sequences from the end of the reads were removed using Trimmomatic^64^. Quality trimmed reads were mapped to *P. falciparum* 3D7 reference genome (release 40 in PlasmoDB- http://www.plasmoddb.org) using BWA (version 0.7.17)^65^. Read pairing information, flag, and duplicate reads were removed using Picard’s CleanSam, FixMateInformation, and MarkDuplicates tools. The full genome sequence has been uploaded in the European Nucleotide Archive. Project ID has been created in ENA with the study accession number PRJEB40003 (Public release date set to 25/08.2021). SNPs were called using the genome analysis tool kit (GATK) best practices pipeline^66^. Identified SNPs were filtered using vcftools to keep high-quality SNP with the quality score (*Q*) ≥ 30 and depth (*d*) ≥50. A total of 105 high-quality SNPs were identified. SNPs were annotated and their effect on coding sequences of genes was done via snpEFF.

For insertion and deletion (InDel) identification, raw gapped alignment were realigned was using GATK RealignerTargetCreator and IndelRealigner tools. Insertion between 20 and 2000 bp and deletion between 20 and 2000 bp were identified using GATK’s SelectVariants tool. Variants were tagged with quality score (*Q*) ≥ 30 and depth (*d*) ≥50 tagged using vcfannotate option and only InDels with a quality score (Q) ≥ 30 and depth (d) ≥100 were filtered for further analysis. Confirmation of absence of the gene sequences of *cas9*, *ampicillin*, *blasticidin*, *hdhfr*, *yfcu*, *flpe recombinase* was determined by concatenating the DNA sequence of these genes into a single fasta file and mapping the quality trimmed reads to the indexed sequences using HISAT2 (V 2.1.0^67^). Read coverage was visualized using Integrative Genome Viewer browser^68^.

### Drug-sensitivity testing of *PfΔmei2*

Drug sensitivity was established in standard drug-sensitivity assays^69^. Comparative drug sensitivity testing was performed for three *P. falciparum* lines (NF54-HGL, WT *Pf*NF54, and *PfΔmei2*) with 7 different compounds in the asexual blood stage (ABS) replication assay. As assay reference, NF54-ΔPf47-5′ hsp70-GFP-Luc^69^ (hereafter: NF54-HGL), a transgenic strain of *P. falciparum* that stably expresses a GFP-Luciferase fusion protein under control of the constitutive *P. falciparum hsp70* promoter, was used. In the assay parasites from asynchronous asexual blood stage cultures were diluted to achieve a parasitemia of 0.8% in 3% hematocrit in RPMI-1640 medium supplemented with 10% human serum. 30 µl of diluted parasites were combined with 30 µl of diluted compound in a 384-well microtiter plate. Following 72 hour incubation at 37 °C, 3% O2, 4% CO_2_, relative parasitemia was determined through a SYBR Green assay. Compounds were dissolved in DMSO to a stock concentration of 10 mM and diluted in DMSO and then in RPMI-1640 medium to reach a final DMSO concentration of 0.1% for all conditions tested. Compounds were tested at 9 dilutions in 0.5 log steps from 10 µM to 1 nM with duplicate wells on each plate. Dihydroartemisinin (DHA) was taken along as a reference compound from 100 pM to 1 µM. Plate controls included MAX (0.1% DMSO) and MIN (1 µM DHA) with 14 replicates each per plate. Relative parasitemia was normalized to the MIN and MAX controls. IC50 values were determined using a four-parameter non-linear regression model using least-squares to find the best fit using the Prism software package (GraphPad Software, San Diego). For each of the plates, Z’ values were calculated based on the MIN and MAX controls.

### Statistical analysis

Statistical analyses were performed using Mann–Whitney test with the GraphPad Prism software package 9 (GraphPad Software, Inc). A *P* value <0.05 was considered significant.

### Reporting summary

Further information on research design is available in the Nature Research Reporting Summary linked to this article.

## Supplementary information

Supplementary Info file

Supplementary Table 1

REPORTING SUMMARY

## Data Availability

Parasite whole-genome sequences have been deposited in the repository https://www.ebi.ac.uk/ena/browser/view/PRJEB40003; the sequence file is accessible under accession number ERR4620262. The *P. berghei* mutant line *Pb*Δ*mei2* has been deposited in the repository https://www.pberghei.eu/index.php?rmgm=4937.
