# Supplementary material for: Creation and preclinical evaluation of genetically attenuated malaria parasites arresting growth late in the liver
Source: NPJ Vaccines. 2022 Nov 4;7:139. doi: 10.1038/s41541-022-00558-x (PMC9636417; doi:10.1038/s41541-022-00558-x)
Supplement: Supplementary file 1 — Supplementary Info file [file 41541_2022_558_MOESM1_ESM.pdf]

Supplementary Figure 1

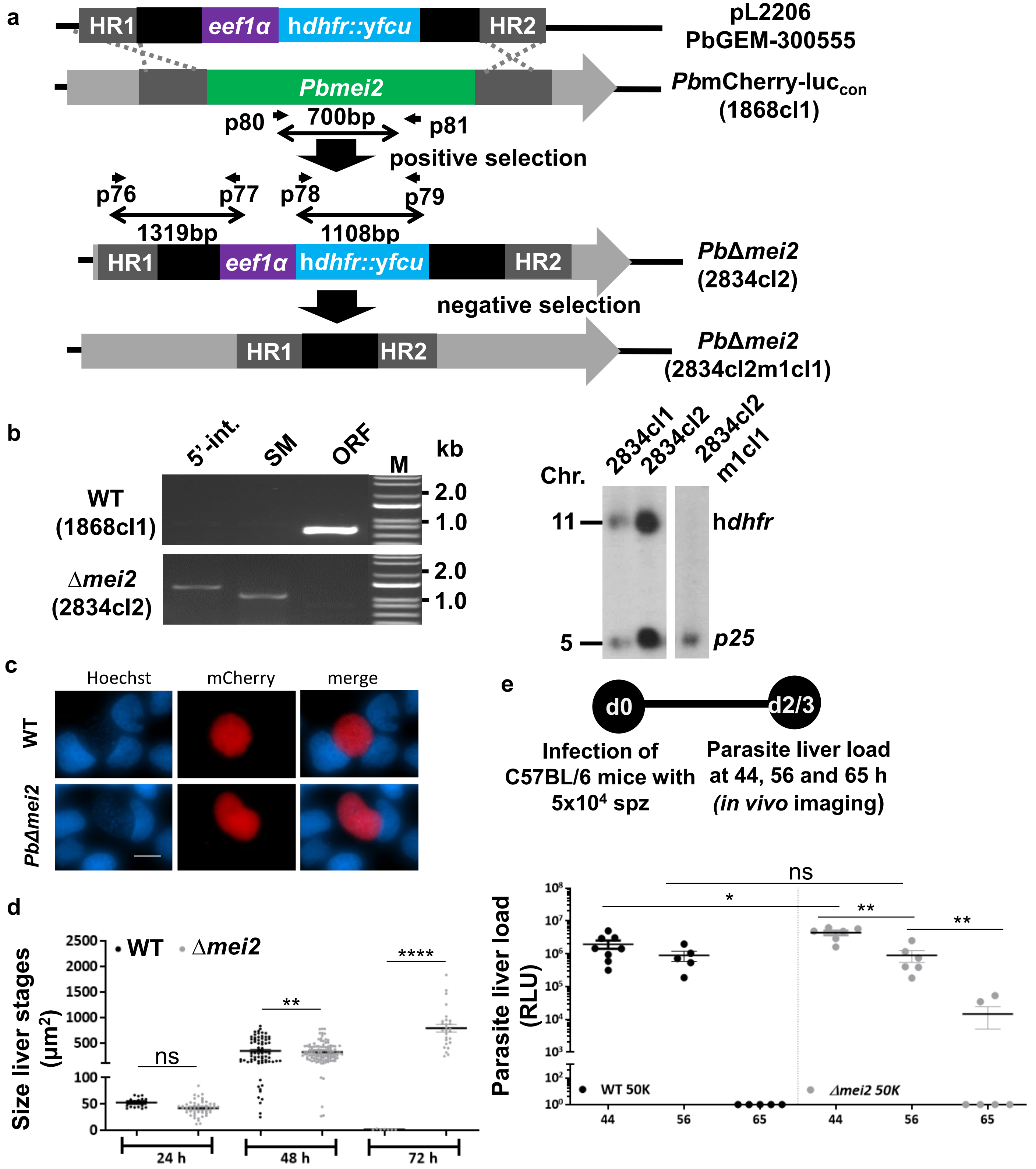

f

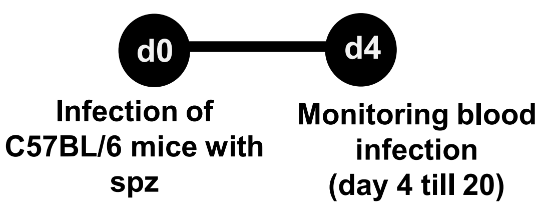

Parasite	No. of spz	No. of mice patent[a]	No. of days to patency[b]
**WT**	5 x 10^3	10/10	5
**WT**	50 x 10^3	10/10	4-5
PbΔmei2	5 x 10^3	0/6	n.a.
PbΔmei2	50 x 10^3	0/6	n.a.
PbΔmei2	200 x 10^3	3/10	9-10
PbΔmei2-b-a	200 x 10^3	5/11	9-10

[a] The number of mice that developed a blood stage infection
[b] The time of blood stage parasitemia between 0.5-2%
WT: wild type; n.a. not applicable

Supplementary Figure 2

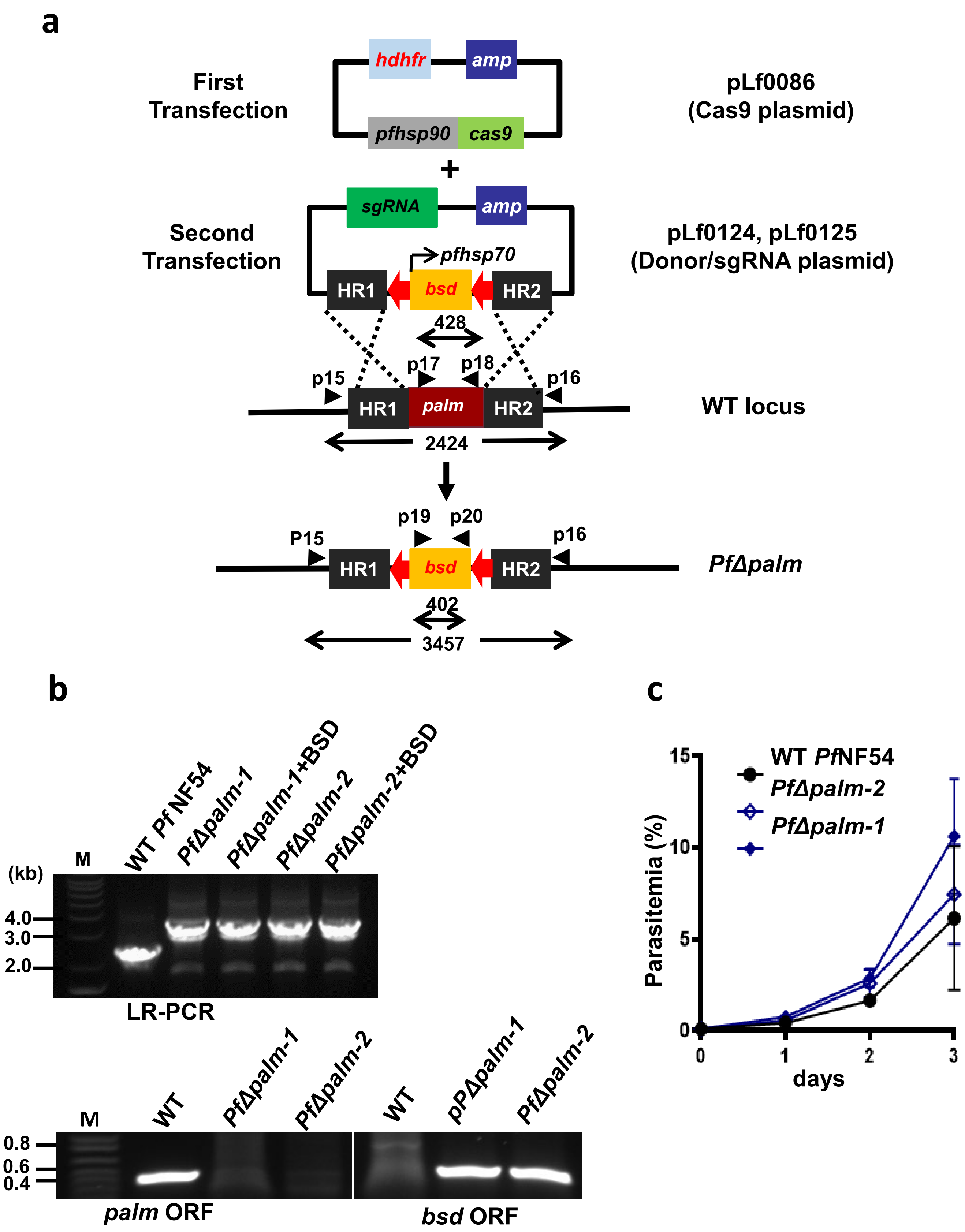

Supplementary Figure 3

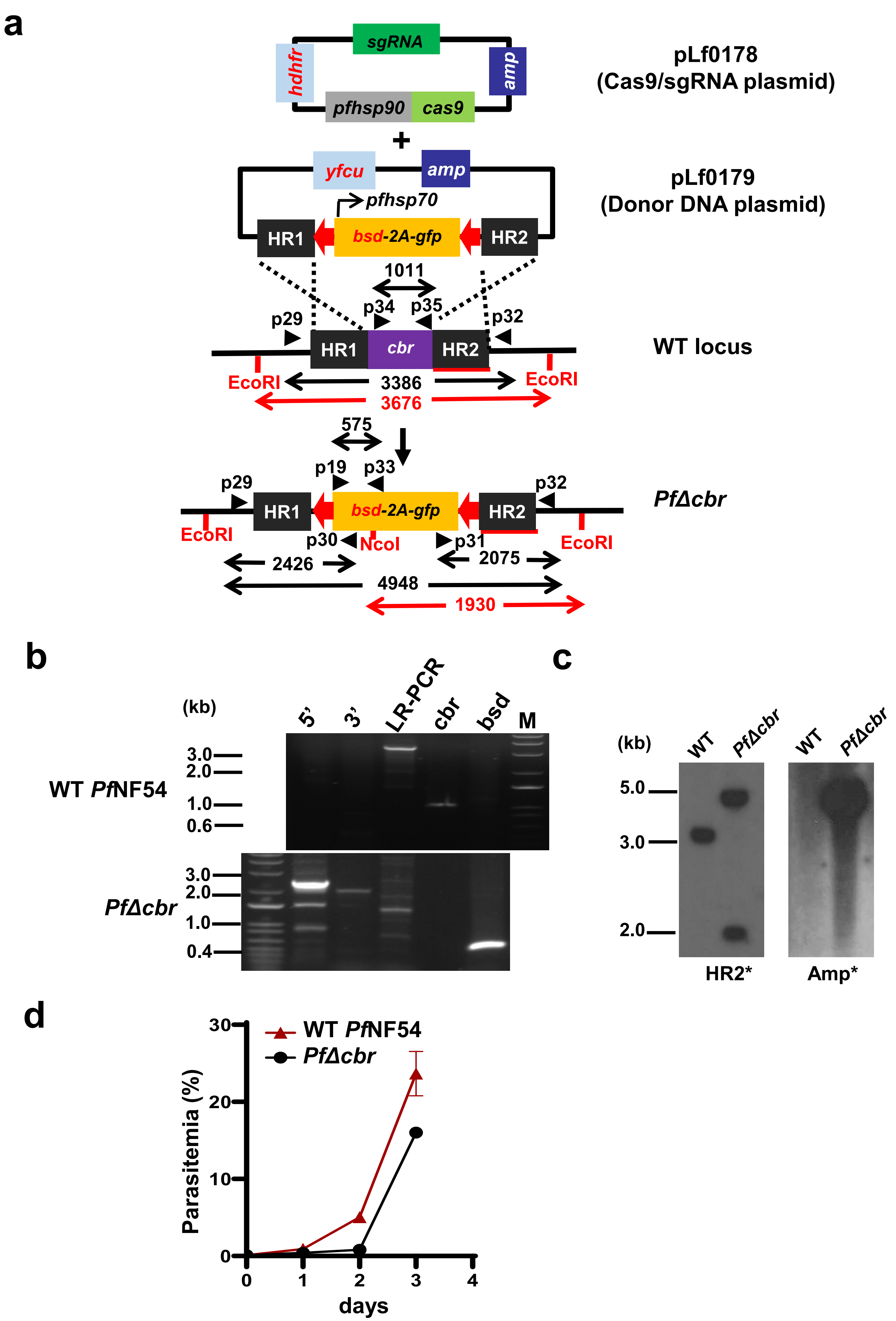

Supplementary Figure 4

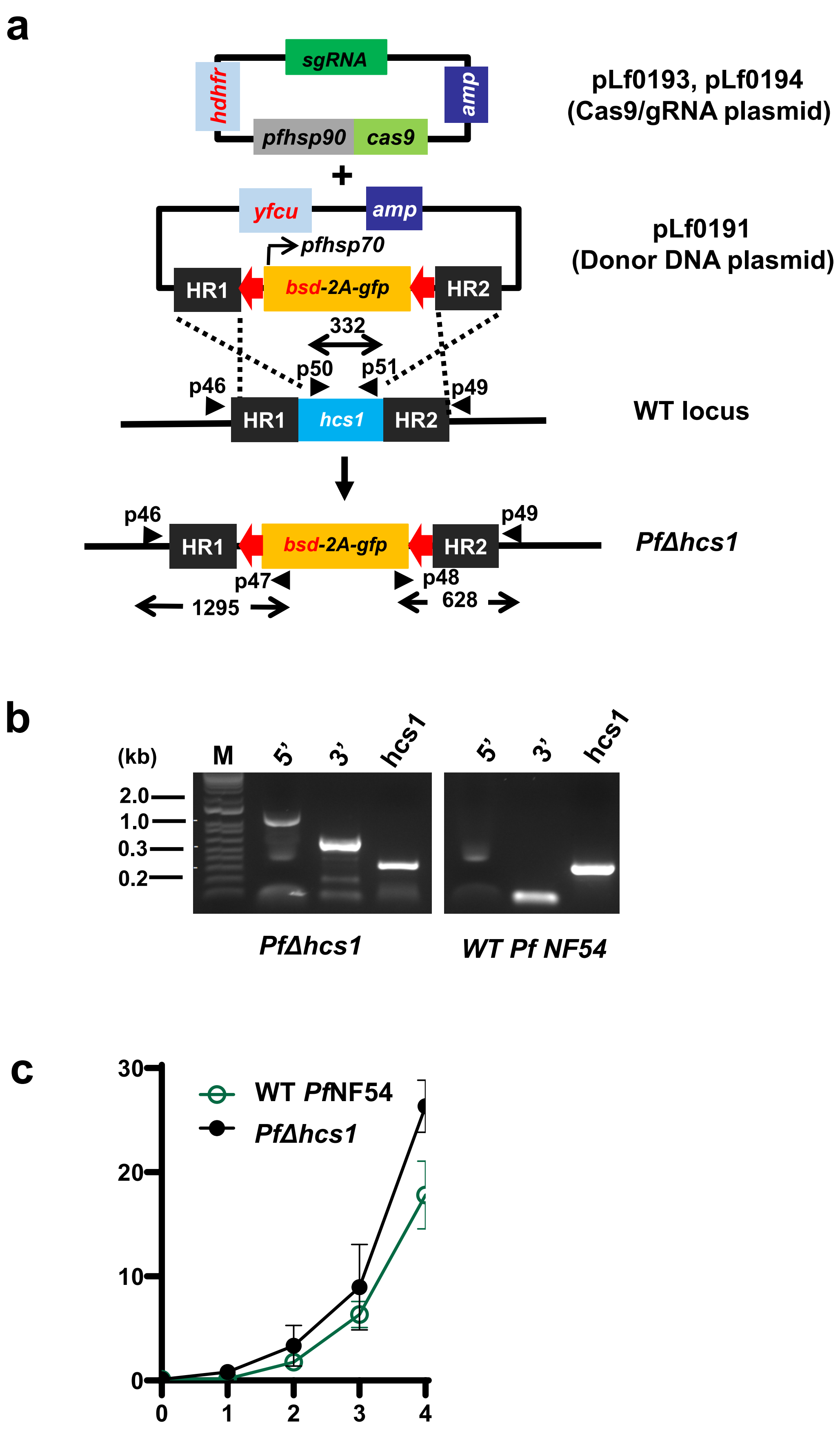

Supplementary Figure 5

WT *Pf*NF54: sporozoite formation

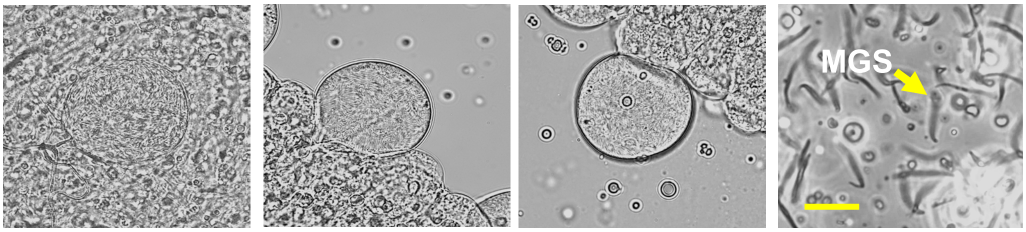

***PfΔmei2:* sporozoite formation**

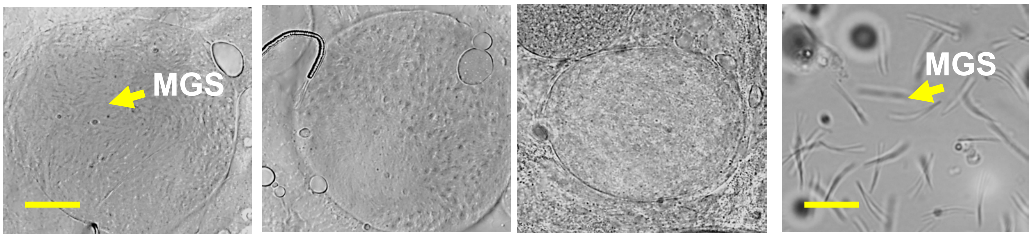

***PfΔpalm, PfΔcbr* and *PfΔhcs1:* Degenerated oocyst (absence of distinct sporozoite formation**

PfΔpalm

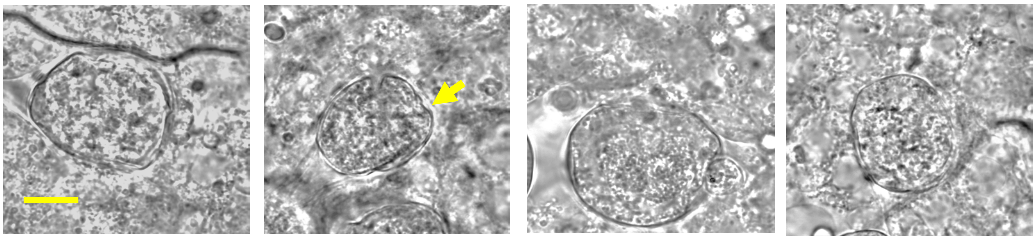

PfΔcbr

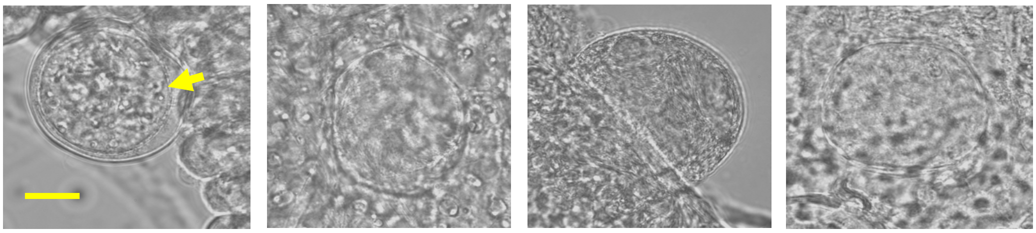

PfΔhcs1

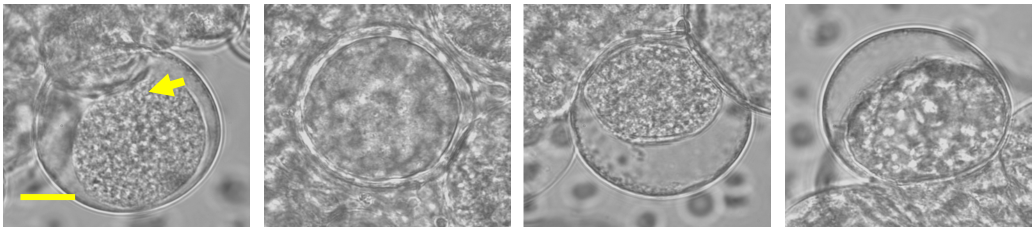

Supplementary Figure 6

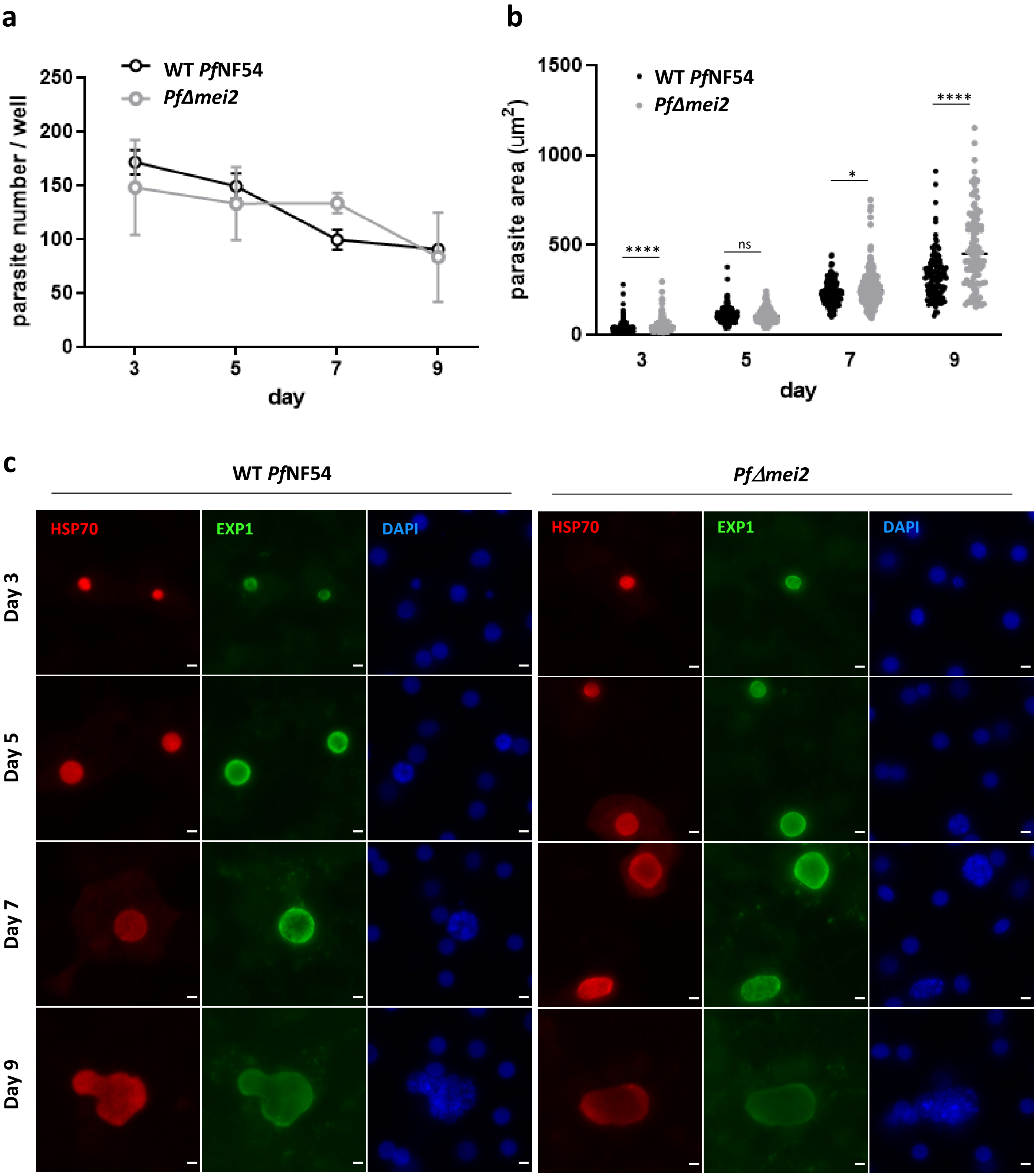

Supplementary Figure 7

a

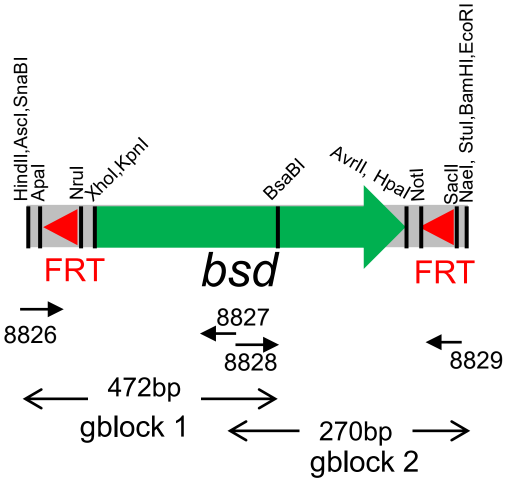

b

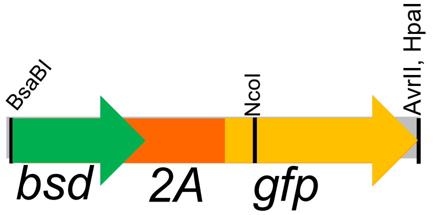

gblock sequence *bsd-2A-gfp*

c **gblock sequence *bsd***

gblock sequence 1

HindIII

TGATTACGCCAAGCTAAGAAG***AAGCTT***gtcagctaGGCGCGCCgtcagctaTACGTAgtcagctaGGGCCCgtcagctaGaagttcct
attctctagaaagtataggaacttcTCGCGAgtcagctaCTCGAGgtcagctaGGTACCgtcagcta**ATG**CATGCCAAGCCTTTGT
CTCAAGAAGAATCCACCCTCATTGAAAGAGCAACGGCTACAATCAACAGCATCCCCATCTCTGAAGACTAC
AGCGTCGCCAGCGCAGCTCTCTCTAGCGACGGCCGCATCTTCACTGGTGTCAATGTATATCATTTTACTG
GGGGACCTTGTGCAGAACTCGTGGTGCTGGGCACTGCTGCTGCTGCGGCAGCTGGCAACCTGACTTGTA
TCGTAGCGATCGGAAATGAGAACAGGGGCATCTTGAGCCCCTGCGGACGGTGCCGACAGGTGCTTCTC***G***
ATCTGCATCCTGGGATCAA

BsaB1

gblock sequence 2

BsaB1

GATCTGCATCCTGGGATCAAAGCCATAGTGAAGGACAGTGATGGACAGCCGACGGCAGTTGGGATTCGT
GAATTGCTGCCCTCTGGTTATGTGTGGGAGGGC**TAA**GTTAACgtcagctaCCTAGGgtcagctaGCGGCCGCgtca
gctaGaagttcctattctctagaaagtataggaacttcgtcaCCGCGGgtcagctaGCCGGCgtcagctaAGGCCTgtcagctaGGATCCgt
cagcta***GAATTC***TTCTTCAATTCACTGGCCGTC

EcoRI

d

gblock sequence *bsd-2a-gfp*

BsaB1

GATCTGCATCCTGGGATCAAAGCCATAGTGAAGGACAGTGATGGACAGCCGACGGCAGTTGGGATTCGTG
AATTGCTGCCCTCTGGTTATGTGTGGGAGGGCGGAGAAGGAAGAGGAAGTTTATTAACATGTGGAGATGTA
GAAGAAAATCCAGGACCA**ATG**AGTAAAGGAGAAGAACTTTTCACTGGAGTTGTCCCAATTCTTGTTGAATTAG
ATGGTGATGTTAATGGGCACAAATTTTCTGTCAGTGGAGAGGGTGAAGGTGATGCAACATACGGAAAACTTA
CCCTTAAATTTATTTGCACTACTGGAAAACTACCTGTTCCATGGCCAACACTTGTCACTACTTTCGCGTATGGT
CTTCAATGCTTTGCGAGATACCCAGATCATATGAAACAGCATGACTTTTTCAAGAGTGCCATGCCCGAAGGTT
ATGTACAGGAAAGAACTATATTTTTCAAAGATGACGGGAACTACAAGACACGTGCTGAAGTCAAGTTTGAAG
GTGATACCCTTGTTAATAGAATCGAGTTAAAAGGTATTGATTTTAAAGAAGATGGAAACATTCTTGGACACAA
ATTGGAATACAACTATAACTCACACAATGTATACATCATGGCAGACAAACAAAAGAATGGAATCAAAGTTAAT
TTCAAAATTAGACACAACATTGAAGATGGAAGCGTTCAACTAGCAGACCATTATCAACAAAATACTCCAATT
GGCGATGGCCCTGTCCTTTTACCAGACAACCATTACCTGTCCACACAATCTGCCCTTTCGAAAGATCCCAA
CGAAAAGAGAGACCACATGGTCCTTCTTGAGTTTGTAACAGCTGCTGGGATTACACATGGCATGGATGAG
CTCTACAAA***CCTAGG***ATT***GTTAACG***TC

AvrII *HpaI*

Supplementary Figure 8

Un-cropped raw images for Figure 1B

PCR gel for Figure 1B

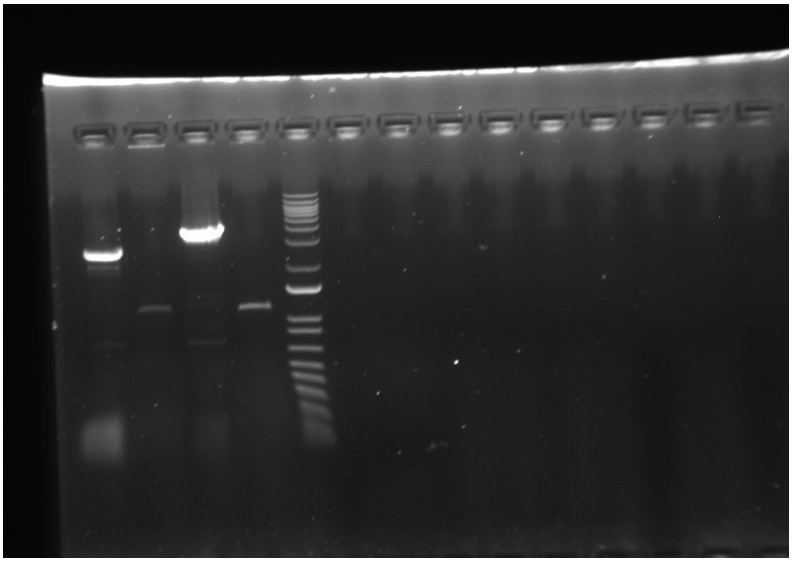

Supplementary Figure 9

Un-cropped raw images for Figure 1C

Gel: 11-11-19
1. -
2. Marker
3. pLf0070
4. pLf0105
5.pLf0070x XmnI
6. pLf0105x XmnI
7. –
8. gDNA 457x XmnI
9. gDNA 491x XmnI
10. –
11. Marker
12. pLf0070
13. pLf0105
14.pLf0070x XmnI
15. pLf0105x XmnI
16. –
17. gDNA 457x XmnI
18. gDNA 491x XmnI
19. –
20. -

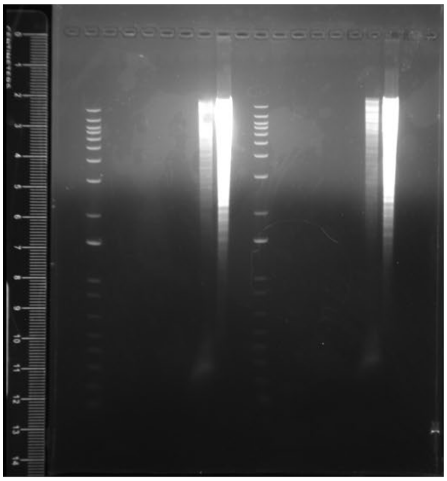

Pf_Southern016
Exposure 1hr
Probe *mei2** Probe *amp*

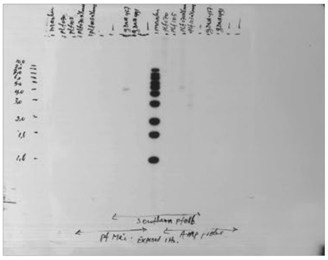

Pf_Southern016
Exposure 2hrs
Probe *p47**

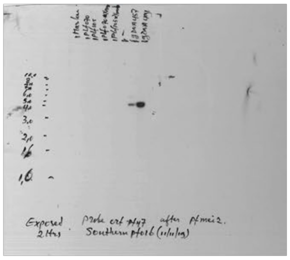

Pf_Southern016
Exposure o/n
Probe *mei2** Probe *amp*

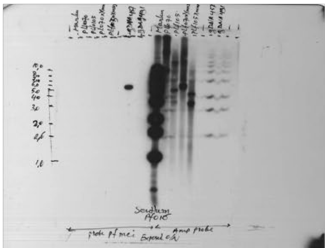

Pf_Southern016
Exposure o/n
Probe *p47*

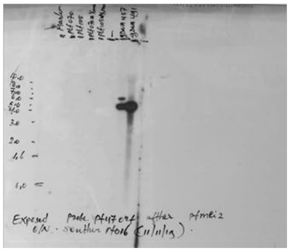

Pf_Southern016
Exposure o/n
Probe *cas9**

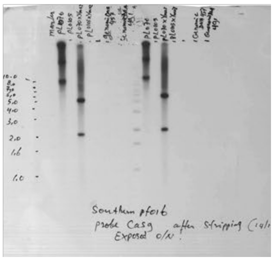

Gel: 30-01-20
1. -
2. Marker
3. pLf0070
4. pLf0105
5.pLf0070x XmnI
6. pLf0105x XmnI
7. –
8. gDNA 457x XmnI
9. gDNA 491x XmnI
10. –
11. Marker
12. pLf0070
13. pLf0105
14.pLf0070x XmnI
15. pLf0105x XmnI
16. –
17. gDNA 457x XmnI
18. gDNA 491x XmnI
19. –
20. -

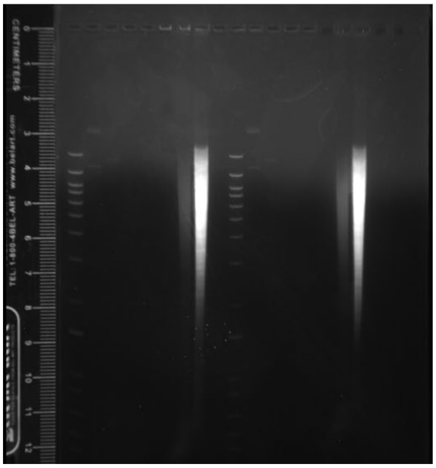

Pf_Southern018
Exposure 2,5hrs
Probe *bsd*

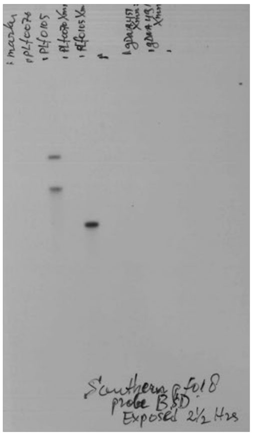

Pf_Southern018
Exposure 5 days
Probe *amp*

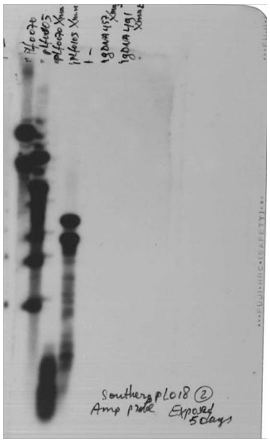

27-7-20
1.Marker
2.pLf120 undig – 40 ng
3.pLf120 dig – 40 ng
4-
5.pLf120 undig – 15 ng
6.pLf120 dig – 15 ng
7.-
8.WT DNA dig
9.Exp.171cl1 DNA dig
10.-
11.Exp.171 uncloned dig

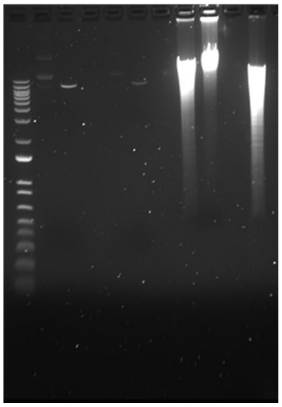

Pf_Southern025
Exposure 5 days
Probe *flpe*

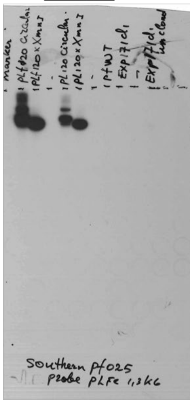

Supplementary Figure 10

Un-cropped raw images for supplementary Figure 1B

PCR gel for Supplementary Figure 1B

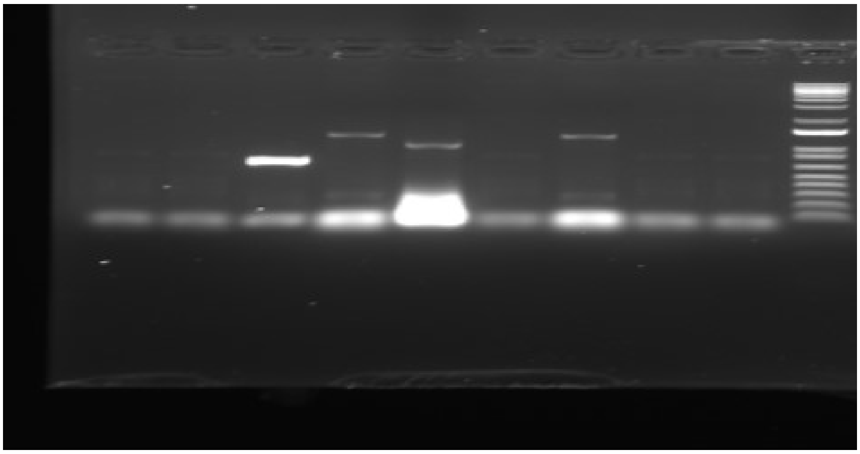

Blots of FIGE gels for Supplementary Figure 1B

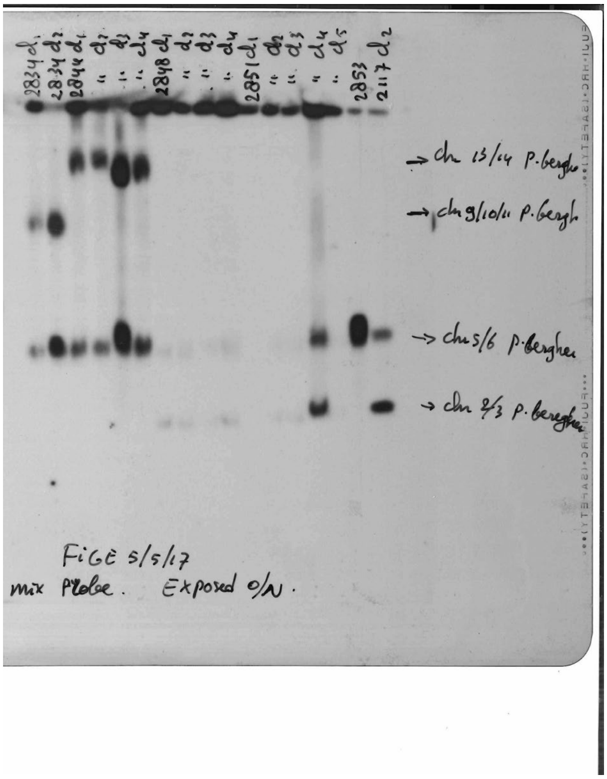

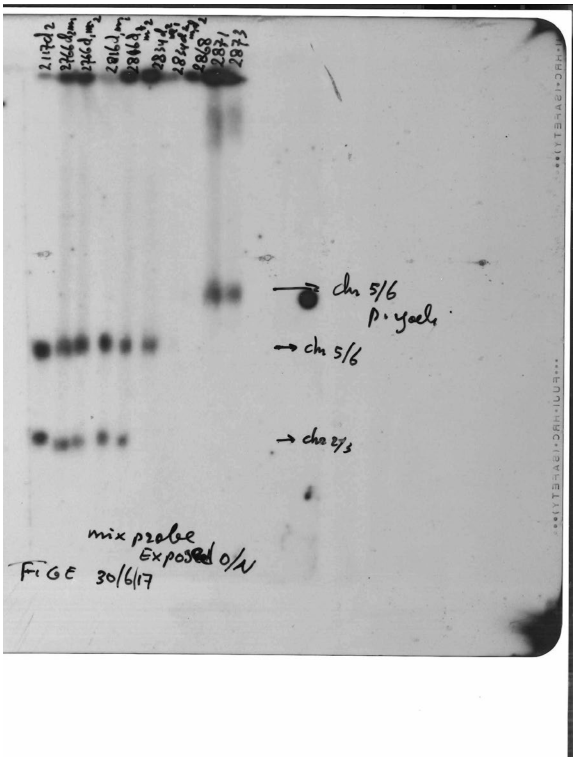

Supplementary Figure 11

Un-cropped raw images of supplementary Figure 2B

PCR gel, upper panel

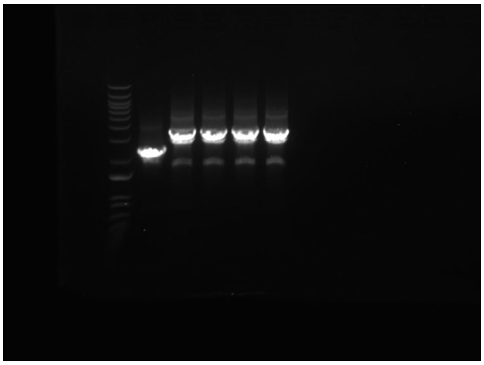

PCR gel, lower panel

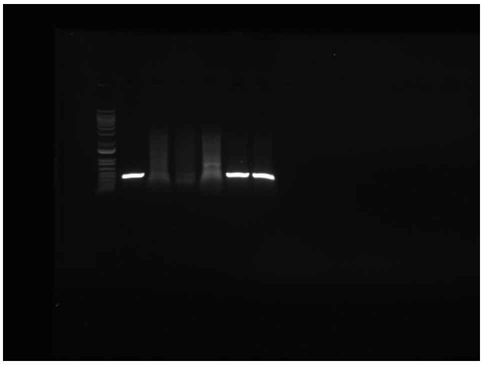

Supplementary Figure 12

Un-cropped raw images of supplementary Figure 3B and 3C

PCR gel, upper panel 3B

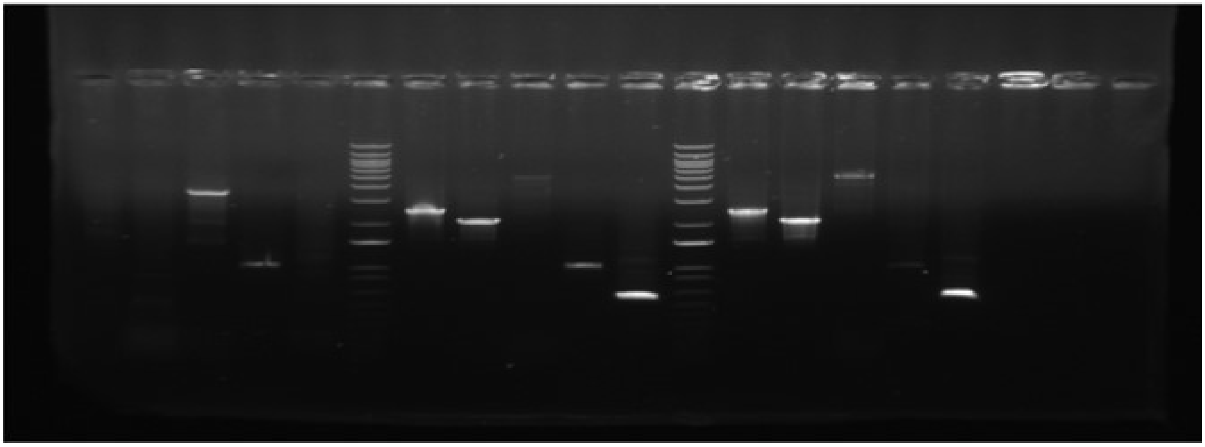

PCR gel, lower panel 3B

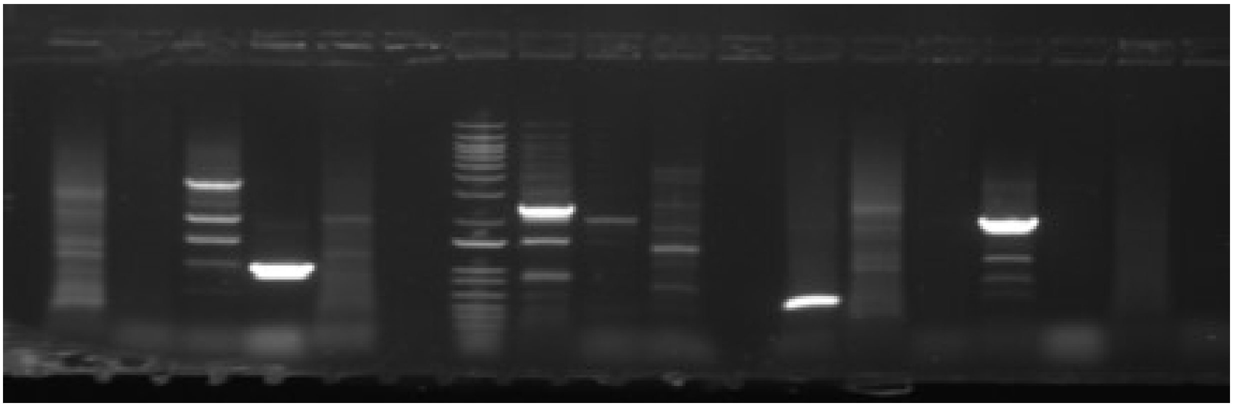

Blot of FIGE gel for Supplementary Figure 3C

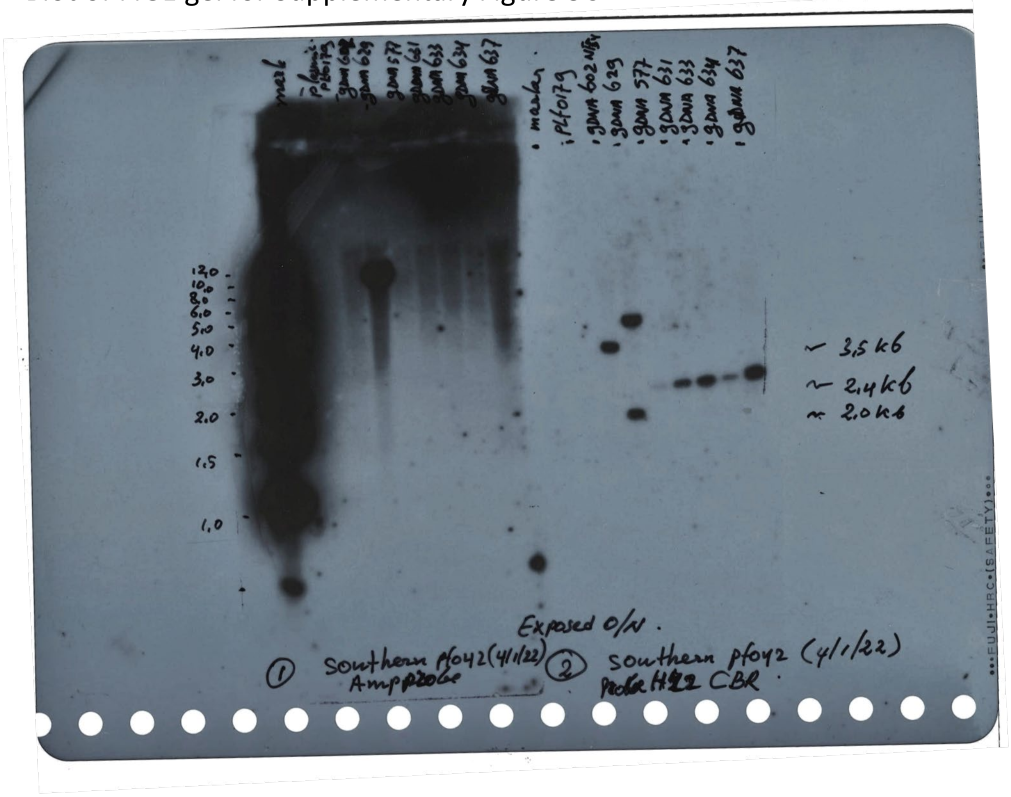

Supplementary Figure 13

Un-cropped raw images of supplementary Figure 4B

PCR gel for Supplementary Figure 4B

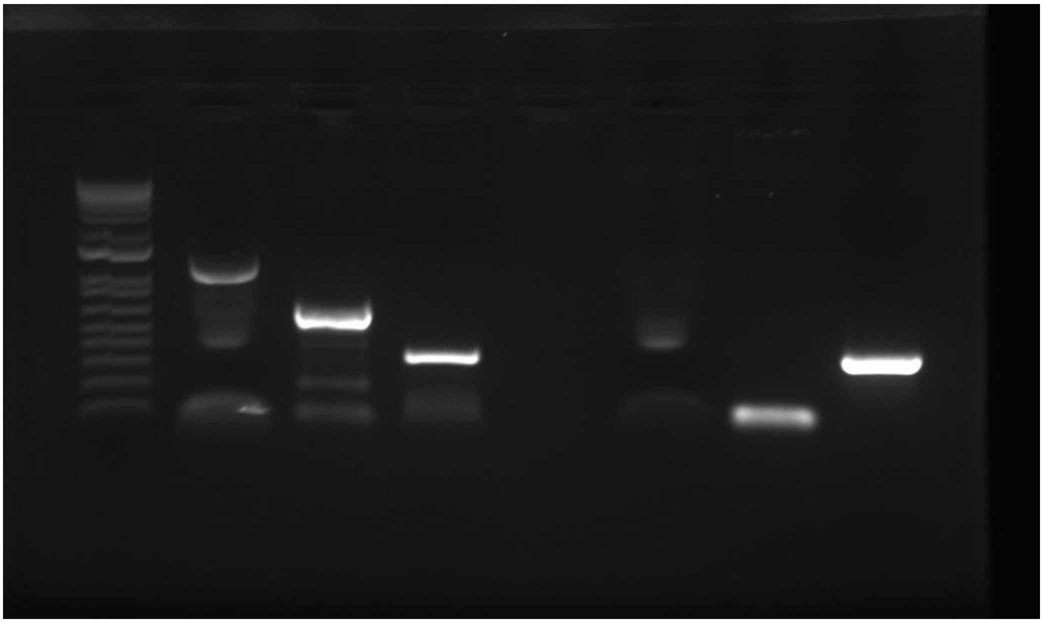

Supplementary Figure legends

Supplementary Figure 1. Generation and genotype and phenotype analysis of the drug-selectable marker free *P. berghei* ANKA gene-deletion mutant *Pb*Δ*mei2*.

a. Schematic representation of the introduction of the h*dhfr*-y*fcu* selectable marker (SM) cassette into the *Pbmei2* gene locus of the parent *P. berghei* parasite (line 1868cl1). Construct pL2206 contains the SM flanked by the *eef1α* promoter region and the 3'-*utr* of pb*dhfr* and integrates into the *mei2* locus by double cross-over homologous recombination at the *mei2* homology regions (HR1, HR2). Positive selection with pyrimethamine selects for parasites that have the *mei2* coding sequence replaced by the SM cassette, resulting in parasite line 2834cl2. To remove the SM cassette from the genome of 2834cl2, blood stage parasites of 2834cl2 were selected (negative selection) by treatment of infected mice with 5-Fluorocytosine (5-FC), selecting for parasites that have undergone homologous recombination between the two 3'-*utr* of pb*dhfr* untranslated regions present in the integrated construct pL2206, flanking the SM cassette and thereby removing the SM. This creates the SM free *Pb*Δ*mei2* gene-deletion mutant (2834cl2m1cl1). Location of primers (p) used for PCR analysis and PCR product sizes are shown. Primer details in **Table S1**.

b. Diagnostic PCR (left panel) and Southern analysis of PFG-separated chromosomes (right panel) confirm correct integration of construct pL2206 in parasites of line 2834cl2. PCR shows the presence of the h*dhfr::*y*fcu* SM (primers **p78/p79**); 5' integration PCR (5'-int; primers **p76/p77**); *mei2* open reading frame PCR (ORF; primers **p80/p81**). Primer locations and product sizes are shown in **A** and primer details in **Table S1**. Hybridization of PFG-separated chromosomes (chr.) (right panel) with a mixture of two probes, h*dhfr* probe and a control probe *p25* (PBANKA_0515000; chromosome 5) shows the presence of the SM in the *mei2* locus on chromosome 11 in two clones of the 2834 line and the absence of the SM in the *mei2* locus on chromosome 11 in SM free *Pb*Δ*mei2* gene-deletion mutant (2834cl2m1cl1). M, molecular weight marker; 1kb DNA ladder (Invitrogen).

c. Representative fluorescence-microscopy images of live parasites at 48 hours after infection of cultures Huh7 hepatocytes with sporozoites of WT and *Pb*Δ*mei2*. Liver-stages express cytoplasmic mCherry under control of the constitutive *hsp70* promoter. Scale bar, 10µm.

d. Size of WT (black) and *Pb*Δ*mei2* (grey) liver-stages at different time points after infection of cultured hepatocytes. The size of parasites (n=20-70) was measured at each time point by determining the area of the parasite's cytoplasm at its greatest circumference using the mCherry-positive area (µm2). Nuclei of parasites and hepatocytes were stained with Hoechst (blue), scale bar: 10 µm. Significance values (unpaired Mann Whitney test): **$p < 0.01$; ****$p < 0.0001$; n.s. – not significant.

e. The time line showing infection of C57BL/6 mice by intravenous injection of 5 × 10(4) of WT (black) and PbΔ*mei2* (grey) sporozoites, followed by determination of parasite liver loads in mice (n=6) by *in vivo* imaging. Parasite liver loads as determined by measuring *in vivo* luciferase activity and depicted as relative light units (RLU). Significance values (unpaired Mann Whitney test): *$p < 0.05$; **$p < 0.01$; n.s. – not significant.

f. The time-line shows infection of C57BL/6 mice with sporozoites and determination of the prepatency of blood infection. The **Table** shows the time to blood-stage patency in C57BL/6 mice infected by intravenous injection of sporozoites.

Error bars represent standard error of the mean.

Supplementary Figure 2. Generation, genotyping and blood-stage growth of *PfΔpalm*

a. Schematic representation (not drawn to scale) of the Cas9 (pLf0086) and the two Donor/gRNA plasmids (pLf0124 and pLf0125) constructs used to generate the two *PfΔpalm* lines (*PfΔpalm*-1, exp. 167 and *PfΔpalm*-2 exp.169). The *Pfpalm* homology regions (HR1, HR2), *blasticidin-S-deaminase* (*bsd*) selectable marker (SM) cassette, location of primers (black arrows) and PCR amplicons (black) are indicated. Primer sequences (in black) are shown in **Table S1**. WT - wild type *Pf*NF54; human *dihydrofolate reductase-thymidylate synthase* (h*dhfr*) SM cassette; yeast cytosine deaminase uridyl phosphoribosyl transferase (y*fcu*) SM cassette; 5' *utr* heat shock protein 90 (*pfhsp90*), *5' utr* heat shock protein 70 (*pfhsp70*), *ampicillin (amp*), single guide RNA (sgRNA).

b. Diagnostic PCR confirms correct integration of the donor/gRNA construct by LR-PCR (primers p15/p16), presence of the *bsd* SM (primers p19/p20) and absence of the *Pfpalm* coding sequence in *PfΔpalm*-1 and *PfΔpalm*-2 parasites (primers p17/p18). Primer locations and product sizes are shown in **(A)** and primer details in **Table S1**. M, molecular weight marker; 1kb DNA ladder (Invitrogen).

c. *In vitro* growth rate of *PfΔpalm*-1, *PfΔpalm*-2 and WT *Pf* NF54 asexual blood-stages. Parasitemia (%) during a 3-day culture period (mean and s.d. of 3 cultures). Error bars represent standard deviation.

Supplementary Figure 3. Generation, genotyping and blood-stage growth of *PfΔcbr*

a. Schematic representation (not drawn to scale) of the Cas9/gRNA plasmid (pLf0178) and the Donor DNA plasmid (pLf0179) construct used to generate the *PfΔcbr* line (exp.236). The *Pfcbr* homology regions (HR1, HR2), the *blasticidin-S-deaminase* (*bsd*) selectable marker (SM) linked to *gfp* through the 2A peptide, location of primers (black arrows) and PCR amplicons (black) are indicated. Primer sequences (in black) are shown in **Table S1**. WT - wild type WT *Pf*NF54; human *dihydrofolate reductase-thymidylate synthase* (h*dhfr*) SM cassette; yeast cytosine deaminase uridyl phosphoribosyl

transferase y*fcu*) SM cassette; 5' *utr* heat shock protein 90 (*pfhsp90*), *5' utr* heat shock protein 70 (*pfhsp70*), *ampicillin (amp*), single guide RNA (sgRNA).

b. Diagnostic PCR confirms correct integration of the donor/gRNA construct by 5' (primers p29/p30) and 3'integration (primers **p31/p32**), presence of the *bsd* SM (primers **p19/p33**) and absence of the *Pfcbr* open reading frame (primers p34/p35) in *PfΔcbr* parasites. Primer locations and product sizes are shown in **(A)** and primer details in **Table S1**. M, molecular weight marker; 1kb DNA ladder (Invitrogen).

c. Southern analysis of digested DNA of WT *Pf*NF54 and *PfΔcbr* confirms *Pfcbr* gene deletion. DNA was hybridized with a probe targeting the HR2 of *Pfcbr* (primers **p31/p32**) and control probe targeting *ampicilin* (*amp*, primers **p36/p37**). The hybridization fragments of WT *Pf*NF54 (3.6 kb) are absent in the *PfΔcbr*, and the two fragments (1.9 and 5.0 kb) of the single crossing-over integration were present in *PfΔcbr*, indicating removal the *Pfcbr* coding sequence by single cross-over integration. Product sizes are shown in **(A).** Primer details in **Table S1**.

d. *In vitro* growth rate of *PfΔcbr* and WT *Pf* NF154 asexual blood-stages. Parasitemia (%) during a 4-day culture period (mean and s.d. of 3 cultures). Error bars represent standard deviation.

Supplementary Figure 4. Generation, genotyping and blood-stage growth *PfΔhcs1*

a. Schematic representation (not drawn to scale) of the Cas9/gRNA plasmids (pLf0193, pLf0194) and the Donor DNA plasmid (pLf0191) construct used to generate the *PfΔhcs1* line (exp.236). The *Pfhcs1* homology regions (HR1, HR2), the *blasticidin-S-deaminase* (*bsd*) selectable marker (SM) linked to *gfp* through the 2A peptide, location of primers (black arrows) and PCR amplicons (black) are indicated. Primer sequences (in black) in **Table S1**. WT - wild type WT *Pf*NF54; human *dihydrofolate reductase-thymidylate synthase* (h*dhfr*) SM cassette; yeast cytosine deaminase uridyl phosphoribosyl

transferase y*fcu*) SM cassette; 5’ *utr* heat shock protein 90 (*pfhsp90*), *5’ utr* heat shock protein 70 (*pfhsp70*), *ampicillin (amp*), single guide RNA (sgRNA).

b. Diagnostic PCR confirms correct integration of the donor/gRNA construct by 5’ (primers **p15/p16**) and 3’ integration (primers **p46/p47**), presence of the *bsd* SM (primers **p48/p49**) and absence of the *Pfhcs1* open reading frame in *PfΔhcs1* parasites (primers **p50/p51**). Primer locations and product sizes are shown in **A** and primer details in **Table S1**. M, molecular weight marker; 1kb DNA ladder (Invitrogen).

c. *In vitro* growth rate of *PfΔhcs1* and WT *Pf* NF154 asexual blood-stages. Parasitemia (%) during a 4-day culture period (mean and s.d. of 3 cultures). Error bars represent standard deviation.

Supplementary Figure 5. Oocyst of WT *Pf*NF54, *PfΔmei2, PfΔpalm, PfΔcbr* and *PfΔhcs1* parasites

a. Light microscope pictures of oocysts from days 9-12 after feeding gametocytes to *An. stephensi* mosquitoes. Sporozoite formation in oocysts from WT *Pf*NF54 and *PfΔmei2* while no sporozoite formation observed in *PfΔpalm, PfΔcbr and PfΔhcs1* oocysts. These oocysts are classified as degenerate based on the absence of distinct sporozoite formation and/or vacuolated cytoplasm. Midgut sporozoites (MGS). Scale bar, 10µm.

Supplementary Figure 6: *Pf*Δ*mei2* liver-stage development in cultured human primary hepatocytes (LONZA)

a. Number of WT *Pf*NF54 and *Pf*Δ*mei2* parasites per well at day3, 5, 7 and 9 p.i. Error bars represent standard error of the mean.

b. Liver-stage size on day 3, 5, 7 and 9 p.i. (99-245 parasites measured in 2 wells). The average of the parasite's cytoplasm at its greatest circumference using HSP70-positive area (μm^2), s.d. and significances values are shown (unpaired Mann Whitney test: $*p < 0.05$; $****p < 0.0001$; ns: not significant).

c. Representative confocal microscopy images of liver-stages on days 3, 5, 7 and 9 p.i. Left panel WT *Pf*NF54; right panel *Pf*Δmei2. Fixed hepatocytes were stained with the following antibodies: mouse anti-*Pf*HSP70 (α hsp70) and rabbit anti-*Pf*EXP1 (α exp1). Nuclei stained with Dapi. All pictures were analyzed using a Cell Insight High Content Screening platform equipped with the Studio HCS software; Alexa Fluor® 488 (green); anti-IgG Alexa Fluor® 594 (red); Dapi (blue). Scale bar, 5µm.

Supplementary Figure 7: Generation of the *bsd* selectable marker cassette of plasmid pLf0103

a. Schematic representation of the *blasticidin-S-deaminase* (*bsd*) selectable marker (SM, in green), flanked by 2 *frt* sites (in red) and restriction sites generated by ligating two gblocks. Primers and PCR fragment are indicated (primer details in **Table S1**).

b. Schematic representation of the *bsd*-2A-*gfp* expression cassette (*bsd* in green, 2A in orange and *gfp* in yellow). Restriction sites are indicated.

c. Sequences of the 2 gblocks used to generate the *bsd* SM (in green), flanked by 2 *frt* sites.

d. Sequence of the gblock used to generate the *bsd*-2A-*gfp* expression cassette (*bsd* in green, 2A in orange and *gfp* in yellow).

Supplementary Figure 8: Raw original images for Figure 1B

Supplementary Figure 9: Raw original images for Figure 1C

Supplementary Figure 10: Raw original images for Supplementary Figure 1B

Supplementary Figure 11: Raw original images for Supplementary Figure 2B

Supplementary Figure 10: Raw original images for Supplementary Figure 3B and 3C

Supplementary Figure 10: Raw original images for Supplementary Figure 4B
