# Supplementary material for: Creation and preclinical evaluation of genetically attenuated malaria parasites arresting growth late in the liver
Source: NPJ Vaccines. 2022 Nov 4;7:139. doi: 10.1038/s41541-022-00558-x (PMC9636417; doi:10.1038/s41541-022-00558-x)
Supplement: Supplementary file 3 — REPORTING SUMMARY [file 41541_2022_558_MOESM3_ESM.pdf]

nature portfolio

Corresponding author(s): Dr Blandine Franke-Fayard

Last updated by author(s): 20-09-2022

Reporting Summary

Nature Portfolio wishes to improve the reproducibility of the work that we publish. This form provides structure for consistency and transparency in reporting. For further information on Nature Portfolio policies, see our Editorial Policies and the Editorial Policy Checklist.

Statistics

For all statistical analyses, confirm that the following items are present in the figure legend, table legend, main text, or Methods section.

n/a	Confirmed	
☐	☒	The exact sample size (*n*) for each experimental group/condition, given as a discrete number and unit of measurement
☐	☒	A statement on whether measurements were taken from distinct samples or whether the same sample was measured repeatedly
☐	☒	The statistical test(s) used AND whether they are one- or two-sided<br>*Only common tests should be described solely by name; describe more complex techniques in the Methods section.*
☐	☒	A description of all covariates tested
☒	☐	A description of any assumptions or corrections, such as tests of normality and adjustment for multiple comparisons
☐	☒	A full description of the statistical parameters including central tendency (e.g. means) or other basic estimates (e.g. regression coefficient) AND variation (e.g. standard deviation) or associated estimates of uncertainty (e.g. confidence intervals)
☐	☒	For null hypothesis testing, the test statistic (e.g. *F*, *t*, *r*) with confidence intervals, effect sizes, degrees of freedom and *P* value noted<br>*Give P values as exact values whenever suitable.*
☒	☐	For Bayesian analysis, information on the choice of priors and Markov chain Monte Carlo settings
☒	☐	For hierarchical and complex designs, identification of the appropriate level for tests and full reporting of outcomes
☒	☐	Estimates of effect sizes (e.g. Cohen's *d*, Pearson's *r*), indicating how they were calculated

Our web collection on statistics for biologists contains articles on many of the points above.

Software and code

Policy information about availability of computer code

Data collection	Images of malaria infected liver cells were taken on a Leica TCS (true confocal scanning) SP5 or SP8X WLL (white light laser) microscope (Leica Microsystems, Wetzlar) and studio HCS software 4.0 (Thermo Fisher Scientific). In vivo imaging data of mice was obtained using LIVING IMAGE 4.1 for the Lumina II from Caliper life sciences.

Data analysis

GA2 genome analysis was performed with the following software:
- P. falciparum 3D7 reference genome (release 40 in PlasmoDB- http://www.plasmoddb.org)
- Burrows-Wheeler transform software; BWA (version 0.7.17)
- quality of the raw reads was assessed using FATSQC (http://www.bioinformatics.babraham.ac.uk/projects/fastqc)
- Low-quality reads and Illumina adaptors sequences from the end of the reads were removed using Trimmomatic
- Picard's CleanSam, FixMateInformation, and MarkDuplicates tools
- genome analysis tool kit (GATK) best practices pipeline (PMID: 20644199)
- Protospacer software (alpha version; https://sourceforge.net/projects/protospacerwb/files/Release/)
Generation of the guide RNA:
- CHOPCHOP webtool (https://chopchop.cbu.uib.no/)
For microscopy analysis:
- Leica LAS X software, Version V 2.5.0
- Studio HCS software 4.0 (Thermo Fisher Scientific)
- GraphPad prism 8.4.3 and 9.3.1
For in vivo imaging data of mice
- LIVING IMAGE 4.1 for the Lumina II from Caliper life sciences
- GraphPad prism 9.3.1

For manuscripts utilizing custom algorithms or software that are central to the research but not yet described in published literature, software must be made available to editors and reviewers. We strongly encourage code deposition in a community repository (e.g. GitHub). See the Nature Portfolio guidelines for submitting code & software for further information.

Data

Policy information about availability of data

All manuscripts must include a data availability statement. This statement should provide the following information, where applicable:

- Accession codes, unique identifiers, or web links for publicly available datasets
- A description of any restrictions on data availability
- For clinical datasets or third party data, please ensure that the statement adheres to our policy

Parasite whole genome sequences have been deposited in the repository https://www.ebi.ac.uk/ena/browser/view/PRJEB40003; the sequence file is accessible under accession number ERR4620262.
The P. berghei mutant line PbΔmei2 has been deposited in the repository https://www.pberghei.eu/index.php?rmgm=4937
Plasmodium falciparum mutant lines generated in this study are available upon request from the corresponding authors (Chris J. Janse, c.j.janse@lumc.nl and B. Franke-Fayard, bfranke@lumc.nl).

Human research participants

Policy information about studies involving human research participants and Sex and Gender in Research.

Reporting on sex and gender

not applicable

Population characteristics

Describe the covariate-relevant population characteristics of the human research participants (e.g. age, genotypic information, past and current diagnosis and treatment categories). If you filled out the behavioural & social sciences study design questions and have nothing to add here, write "See above."

Recruitment

Describe how participants were recruited. Outline any potential self-selection bias or other biases that may be present and how these are likely to impact results.

Ethics oversight

Identify the organization(s) that approved the study protocol.

Note that full information on the approval of the study protocol must also be provided in the manuscript.

Field-specific reporting

Please select the one below that is the best fit for your research. If you are not sure, read the appropriate sections before making your selection.

☒ Life sciences ☐ Behavioural & social sciences ☐ Ecological, evolutionary & environmental sciences

For a reference copy of the document with all sections, see nature.com/documents/nr-reporting-summary-flat.pdf

Life sciences study design

All studies must disclose on these points even when the disclosure is negative.

Sample size

No statistical test was used to predetermine sample size. Instead, sample sizes were rationalized by considering sufficient replication (weighing the level of biological variation)

Data exclusions	All data were included in analyses
Replication	The parasites mutants (mei2, cbr, pal, hcs1) were generated in duplicate dependent experiments for rodent malaria parasite and for the human malaria parasites.<br>The phenotype analyses (mosquito experiments and liver stage experiments) were replicated in multiple independent experiments.<br>Drug testing assay: The NF54wt and GAP strains were supplied as culture replicates and each of the concentrations tested was measured in quadruplicate.
Randomization	Sample allocation was not random. Instead, biological controls were employed in all experiments
Blinding	Blinding was not performed in all the experiments due to distinct phenotypes of different mutant parasite lines.

Reporting for specific materials, systems and methods

We require information from authors about some types of materials, experimental systems and methods used in many studies. Here, indicate whether each material, system or method listed is relevant to your study. If you are not sure if a list item applies to your research, read the appropriate section before selecting a response.

Materials & experimental systems

n/a	Involved in the study
☐	☒ Antibodies
☐	☒ Eukaryotic cell lines
☒	☐ Palaeontology and archaeology
☐	☒ Animals and other organisms
☒	☐ Clinical data
☒	☐ Dual use research of concern

Methods

n/a	Involved in the study
☒	☐ ChIP-seq
☒	☐ Flow cytometry
☒	☐ MRI-based neuroimaging

Antibodies

Antibodies used	- the cytoplasmic protein PfHSP70 (PF3D7_0818900; rabbit anti-PfHSP70-PE/ATTO 594 conjugated primary antibody (Catalog No. SPC-186, StressMarq, Biosciences<br>- polyclonal anti-PfHSP70 murine serum prepared in house, ICM, La Pitié-Salpêtière, Paris, France<br>- the plasma membrane surface protein MSP1 (PF3D7_0930300), mouse monoclonal antibody obtained from The European Malaria Reagent Repository, Edinburgh, UK<br>- the parasitophorous membrane protein EXP1 (PF3D7_1121600, mouse monoclonal antibody obtained from The European Malaria Reagent Repository, Edinburgh, UK<br>- rabbit anti-PfEXP1 from Pr Jude Przyborski<br>- Alexa-Fluor 488-conjugated goat anti-mouse immunoglobulin (Molecular probes; A11001)<br>-Alexa-Fluor 488-conjugated goat anti-rabbit immunoglobulin (Invitrogen)<br>- anti-mouse IgG Alexa Fluor® 594 (Invitrogen)
Validation	Primary and secondary antibodies used in this study are well accepted in the field and purchased from reputable suppliers with provided quality control metrics (except polyclonal anti-PfHSP70 murine serum and rabbit anti-PfEXP1 generated at CM, La Pitié-Salpêtière, Paris, France, those antibodies have been used in previous peer review studies).

Eukaryotic cell lines

Policy information about cell lines and Sex and Gender in Research

Cell line source(s)	P. berghei ANKA parasite line 1868cl1 generated in the lab that expresses mCherry and luciferase under the constitutive hsp70 and eef1a promotors, respectively (RMgm-1320, www.pberghei.eu). The PfNF54 strain was isolated in 1979 from a Dutch malaria patient and was first adapted to continuous in vitro culture system. Parasites from the PfNF54 strain, and its derivative Pf3D7, are the most commonly used Pf parasites in laboratory studies. The complete genome sequences of Pf3D7 and PfNF54 has been published. The parasites of PfNF54 and Pf3D7 have been deposited with the Malaria Research and Reference Reagent Resource Center (MR4; MRA-1000 and MRA-102), which was developed by the National Institute of Allergy and Infectious Diseases (NIAID) and is managed by the American Type Culture Collection (ATCC) (BEI Resources; https://www.beiresources.org/About/MR4.aspx).<br>Huh7 (JCRB0403), human hepatoma cell line was obtained from JCRB Cell Bank, Japan.<br>Human preserved hepatocytes were purchased from Lonza Bioscience and BioIVT.
Authentication	The different P. berghei ANKA lines and P. falciparum NF54 lines were confirmed by genotyping (PCR, qPCR and Southern analysis).
Mycoplasma contamination	The cell lines were not tested for mycoplasma contamination

Commonly misidentified lines (See ICLAC register)	No commonly misidentified cell lines were used in this study

Animals and other research organisms

Policy information about studies involving animals; ARRIVE guidelines recommended for reporting animal research, and Sex and Gender in Research

Laboratory animals	Female OF1 mice (6-7 weeks, Charles River Laboratories, NL), female C57Bl6 mice (6-7 weeks from Charles River Laboratories, USA) and Liver-chimeric humanized mice (FRG huHep mice) purchased from Yecuris Corporation (Tualatin, OR) were used.
Wild animals	The study did not involve wild animals.
Reporting on sex	Findings do not apply to only one sex.
Field-collected samples	No field-collected samples were used in this study
Ethics oversight	All animal experiments were granted with a license by Competent Authority after an advise on the ethical evaluation by the Animal Experiments Committee Leiden (DEC12042 and 14207). All experiments were performed in accordance with the Experiments on Animals Act (Wod, 2014), the applicable legislation in the Netherlands in accordance with the European guidelines (EU directive no. 2010/63/EU). All experiments were executed in a licensed establishment for the use of experimental animals (LUMC).<br>All studies involving Liver-chimeric humanized mice (FRG huHep mice) were performed at Oregon Health and Science University (OHSU) according to the regulations of the Institutional Animal Care and Use Committee (IACUC; protocol IP00002077).

Note that full information on the approval of the study protocol must also be provided in the manuscript.
